# Complete mitochondrial genomes from transcriptomes: assessing pros and cons of data mining for assembling new mitogenomes

**DOI:** 10.1038/s41598-019-51313-7

**Published:** 2019-10-15

**Authors:** Giobbe Forni, Guglielmo Puccio, Thomas Bourguignon, Theodore Evans, Barbara Mantovani, Omar Rota-Stabelli, Andrea Luchetti

**Affiliations:** 1 Department of Biological, Geological and Environmental Sciences - University of Bologna, via Selmi 3, 40126 Bologna, Italy; 2 Okinawa Institute of Science & Technology Graduate University, 1919-1 Tancha, Onna-son, Okinawa 904–0495 Japan; 3 Faculty of Forestry and Wood Sciences, Czech University of Life Sciences, Prague, Czech Republic; 4 School of Animal Biology, University of Western Australia, Perth, WA 6009 Australia; 5 Agrarian Entomology, Research and Innovation Centre, Fondazione Edmund Mach (FEM), Via E. Mach 1, 38010 San Michele all’Adige, TN Italy

**Keywords:** Data mining, Molecular evolution, Phylogenetics, Transcriptomics

## Abstract

Thousands of eukaryotes transcriptomes have been generated, mainly to investigate nuclear genes expression, and the amount of available data is constantly increasing. A neglected but promising use of this large amount of data is to assemble organelle genomes. To assess the reliability of this approach, we attempted to reconstruct complete mitochondrial genomes from RNA-Seq experiments of *Reticulitermes* termite species, for which transcriptomes and conspecific mitogenomes are available. We successfully assembled complete molecules, although a few gaps corresponding to tRNAs had to be filled manually. We also reconstructed, for the first time, the mitogenome of *Reticulitermes banyulensis*. The accuracy and completeness of mitogenomes reconstruction appeared independent from transcriptome size, read length and sequencing design (single/paired end), and using reference genomes from congeneric or intra-familial taxa did not significantly affect the assembly. Transcriptome-derived mitogenomes were found highly similar to the conspecific ones obtained from genome sequencing (nucleotide divergence ranging from 0% to 3.5%) and yielded a congruent phylogenetic tree. Reads from contaminants and nuclear transcripts, although slowing down the process, did not result in chimeric sequence reconstruction. We suggest that the described approach has the potential to increase the number of available mitogenomes by exploiting the rapidly increasing number of transcriptomes.

## Introduction

The NCBI GenBank database counts more than 78,000 mitochondrial genome entries from more than 30,000 different species. Due to their relatively small size, ease of sequencing and clear orthology, mitochondrial genomes are currently the most widely used genomic markers for animal systematics and phylogenetic studies, particularly in insects^1^. Mitogenome-based phylogenies have helped resolving inter-ordinal^2^, intra-ordinal^3^ and intra-familial^4,5^ relationships. Many biogeographic, population genetics, and museum genomics studies also rely on mitochondrial genome sequencing^6,7^.

Most mitochondrial genomes are now obtained with high-throughput sequencing (HTS) of DNA (either including a long PCRs step or by direct HTS sequencing of total DNA) and a wide variety of methods have been developed for sequencing, assembly and annotation^8,9^. As pointed out by Smith^10^, a considerable amount of information for organelle genomics may be obtained from RNA-Seq data and, taking into account the rate at which transcriptome data accumulate, they can be further used to recover mitochondrial genomes from taxa in which they are not available yet. The NCBI SRA database (Sequence Read Archive; last accessed January 2019) stores transcriptome raw reads of more than 1,790 different insect species: for about 1,000 of these species there are no mitogenomes in the Nucleotide archive (last accessed January 2019). Considering that more than 4,800 insect mitogenomes are actually present in Genbank databases, mining mitochondrial genomes from transcriptomes has the potential to increase the number of insects mitogenomes by ~20%.

The use of RNA-Seq data for mitochondrial genomes reconstruction provides several advantages. Genes from organelles have higher expression levels than nuclear genes and, therefore, a large portion of raw reads generated from eukaryotic RNA-Seq experiments are of organelle origin^11^. Because organelle genomes are pervasively transcribed as polycistronic RNAs, it is possible to recover almost complete mitogenomes from transcriptomes^12^. On the other hand, the use of RNA-Seq data could still retain some of the drawbacks of DNA-based approaches. As an example, despite the low chance of a NUMT (nuclear mitochondrial DNA; *i.e*. mitochondrial DNA copies migrated into the nuclear genome) to be integrated in a nuclear transcript, there are some evidences of the presence of nuclear mitochondrial pseudogenes in transcriptomic data^13^.

Nonetheless, literature indicates that transcriptome data is not yet routinely used to assemble mitogenomes. A few studies, for example, report on successfully assembled mitochondrial genomes either by mapping reads on conspecific references and assembling them^14,15^, or by mining mitochondrial contigs from de novo assembled transcriptomes^16,17^. However, most of these studies did not provide new mitochondrial genomes and, in more general terms, all of them were unable to assemble regions, such as tRNAs or the control region, due to low reads coverage. This latter finding, besides being of fundamental importance to obtain fully assembled mitogenomes, suggests that some pitfalls may occur when dealing with RNA-Seq data for mitogenomic studies.

In this report we investigate potential pros and cons of de novo assembling mitogenomes from transcriptome data in order to get information about genome structure and nucleotide variability, in a systematic framework. For this aim, we mined mitochondrial DNA from transcriptome data of the subterranean termite genus *Reticulitermes* (Blattodea; Termitoidae) as a case study. RNA-Seq data and mitogenome sequences are already available for several species of *Reticulitermes*, allowing direct comparison of transcriptome-derived mitogenome sequences with those obtained through traditional methods (long-PCR + Sanger sequencing or high throughput genome sequencing). Moreover, we assembled for the first time the mitochondrial genome of *Reticulitermes banyulensis*. The genus *Reticulitermes* has been the subject of many phylogenetic studies during the last two decades^18,19^, making it a suitable group to test the phylogenetic accuracy of the mitogenomes reconstructed from transcriptomes.

Overall, we demonstrate the validity of the iterative reference mapping and de novo assembly of mitogenomes from transcriptomes, recovering reliable complete molecules from the assayed species. On the other hand, some pitfalls may emerge, such as in the case of contaminants, similarity with nuclear transcripts or for tRNA reconstruction.

## Results and Discussion

### Reconstruction process analysis

Overall, the reconstruction process led to the assembly of either complete or nearly complete mitogenomes for all the six analyzed species (Supplementary Fig. S1).

**Figure 1:**
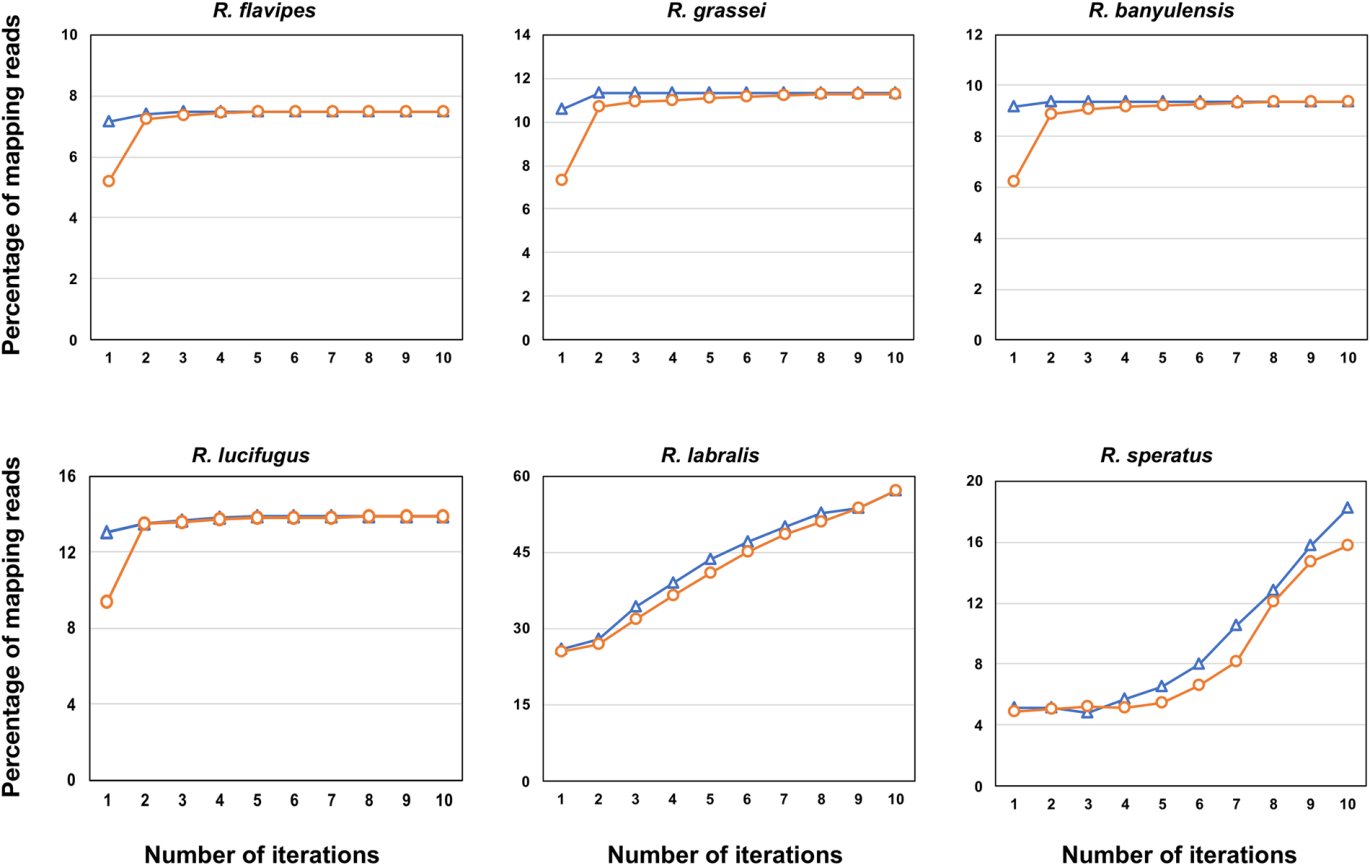
Short reads recruitment across iterations. Relationships between the number of iterations of the process (*x* axis) and mapped reads (% of the total reads; *y* axis) using congeneric (blue triangles) or intra-familial (orange circles) references.

**Figure 2:**
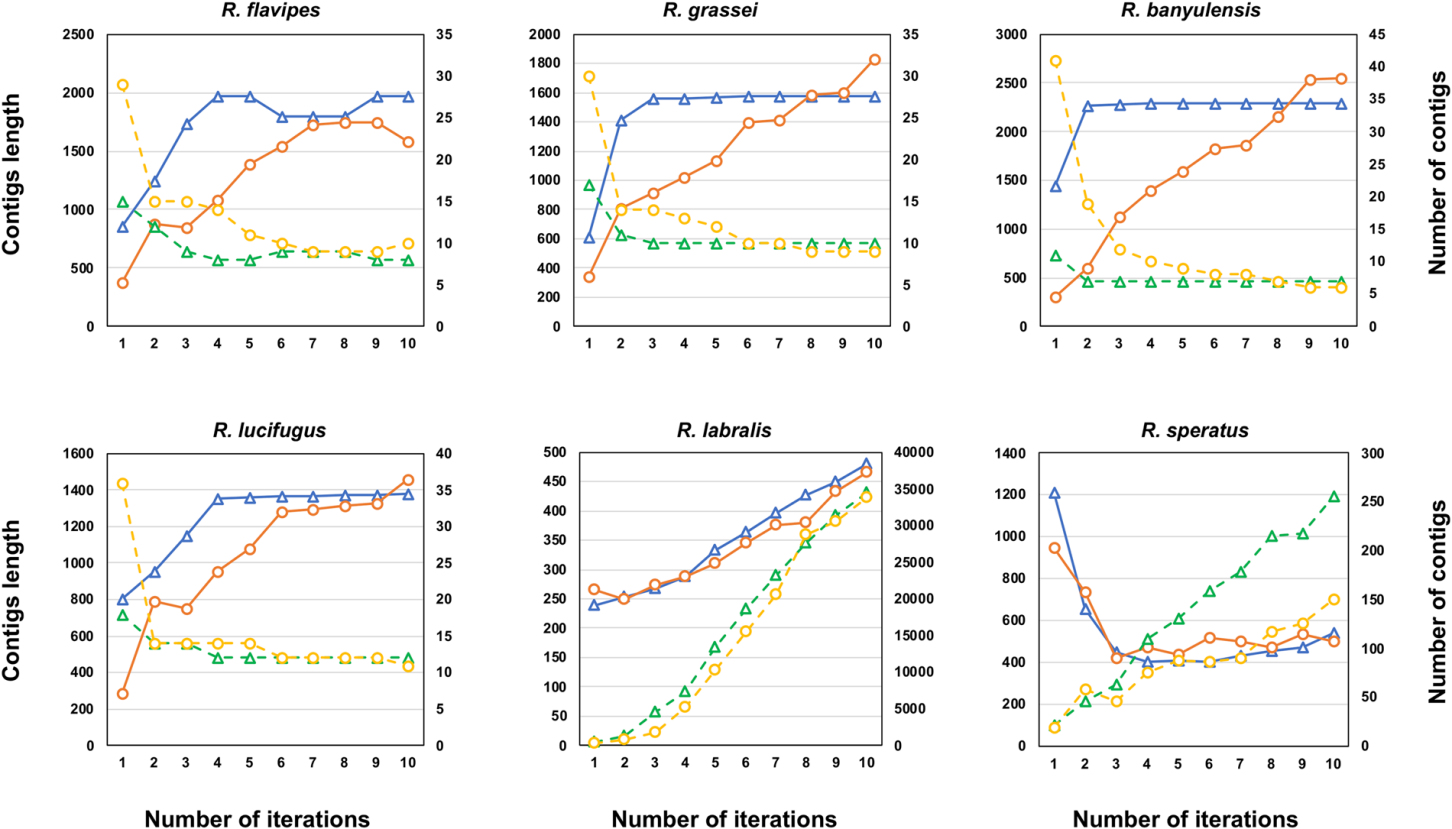
Contigs analysis across iterations. Relationship between the number of iteration process (*x* axis) and contigs length (bp, primary *y* axis; solid lines) and contigs number (secondary *y* axis; dashed lines) using congeneric (blue triangles) or intra-familial (orange circles) references.

For *R. flavipes*, *R. grassei*, *R. banyulensis* and *R. lucifugus*, the number of iterations necessary to mine all mitochondrial reads (i.e., in our pipeline, to reach a stationary number of mapping reads) varied depending on the phylogenetic relatedness of the initial reference (congeneric as blue triangles; intra-familial as orange circles in Fig. 1). As it could be expected, the use of intra-familial reference led to recover fewer reads during the first iterations than using congeneric references (Fig. 2). However, all assemblies, whether based on congeneric or on intra-familial references, converged to a plateau within the 10^th^ iteration; at this point, the total number of mitochondrial reads covered between 7.5% and 13.9% of the whole transcriptomes. We observed fewer and longer contigs as the number of iterations increased (Fig. 2) and this trend was not influenced by the choice of the initial reference. Overall, the number of contigs at the 10th iteration varied between six and twelve, and their average length varied between 1,376 and 2,544 bp. In contrast, no plateau was reached within the 10th iteration for both *R. labralis* and *R. speratus*, independently of the initial references (Fig. 1). With respect to the other four species, the iteration process for *R. labralis* and *R. speratus* resulted in a far larger number of contig (33,906–34,439 and 157–262, respectively; Table 1). Moreover, we did not observe a reduction in number and an increase in length of the contigs throughout the iterations (Fig. 2). In *R. labralis*, the percentage of recruited reads reached 57%, with both intra-familial and congeneric starting references. In *R. speratus*, the percentage of recruited reads reached 18% and 16% using the intra-familial and congeneric starting references, respectively. We analyzed more in details the contigs obtained after the 10th iteration in these two species, filtering for possible contaminants and nuclear genes matches. In both species, mitochondrial contigs represented the minority of assembled ones: less than the 1% in *R. labralis* and less than the 14% in *R. speratus*. The majority of contigs were, therefore, contaminant or nuclear transcripts, with a different relative contribution of the two kinds of leakage in the two species (Table 1). The contaminant leakage played a major role in *R. labralis*, with fraction of contigs significantly matching also to sequences of non-hexapod taxa (Table 1 and Suppl. Table S1). On the other hand, the nuclear leakage appeared predominant in *R. speratus* (Table 1 and Suppl. Table S1).

**Table 1:** Non-mitochondrial reads leakage analysis in *Reticulitermes labralis* and *R. speratus* RNA-Seq experiments.

Contigs at the 10^th^ iteration	*Reticulitermes labralis*		*Reticulitermes speratus*	
	Congeneric references	Intra-familial references	Congeneric references	Intra-familial references
Total	34,439	33,906	262	157
Target mitochondrial contigs	138 (0.40%)	180 (0.53%)	32 (12.2%)	22 (14.0%)
Contaminant leakage	23,819 (69.2%)	23,572 (69.5%)	113 (43.1%)	66 (42.0%)
Nuclear leakage	10,482 (30.4%)	10,154 (29.9%)	117 (44.7%)	69 (43.9%)

Numbers refer to contigs number (percentage) that were found of contaminant or nuclear DNA origin.

Excluding contaminant and nuclear transcripts, all contigs obtained at the end of the automated process in the six assayed species matched the relative mitochondrial genomes. On the other hand, a few gaps were still present at the end of the iterative reconstruction process. These gaps encompassed mainly tRNA regions and/or the control region, with the only exception of a fragment of the *R. speratus atp6* gene (Supplementary Fig. S1a). Only *R. banyulensis* and *R. labralis* resulted completely reconstructed (except the control region) at the end of the iterative process. The use of intra-familial references in the first iteration, instead of congeneric ones, led to similar results but with slightly larger gap regions and no mitogenomes completely reconstructed at the end of the process (Supplementary Fig. S1b). We recovered missing regions using *blastn* search of the transcriptome reads against the homolog portion of the reference mitochondrial genomes and filled the gaps. Control regions remained only partially assembled, mainly because they are composed of tandem repeats, thus very difficult to de novo assemble properly, and their expression is low or completely lacking. Moreover, we obtained partial control regions only when mitochondrial genomes of congeneric species were used as initial references (Supp. Table S2).

### Mitogenomes completeness and accuracy assessment

The six mitochondrial genomes we recovered were comparable in length and content to that of other insect mitogenomes, containing 13 PCGs, 22 tRNAs and 2 rRNAs, with gene order consistent to that of other *Reticulitermes* mitochondrial genomes. No gap or stop codon were detected within open reading frames and no significant variation of tRNA cloverleaf structures was found between the RNA-Seq assembled mitogenomes and those already available from DNA sequencing (Supplementary Fig. S2).

**Figure 3:**
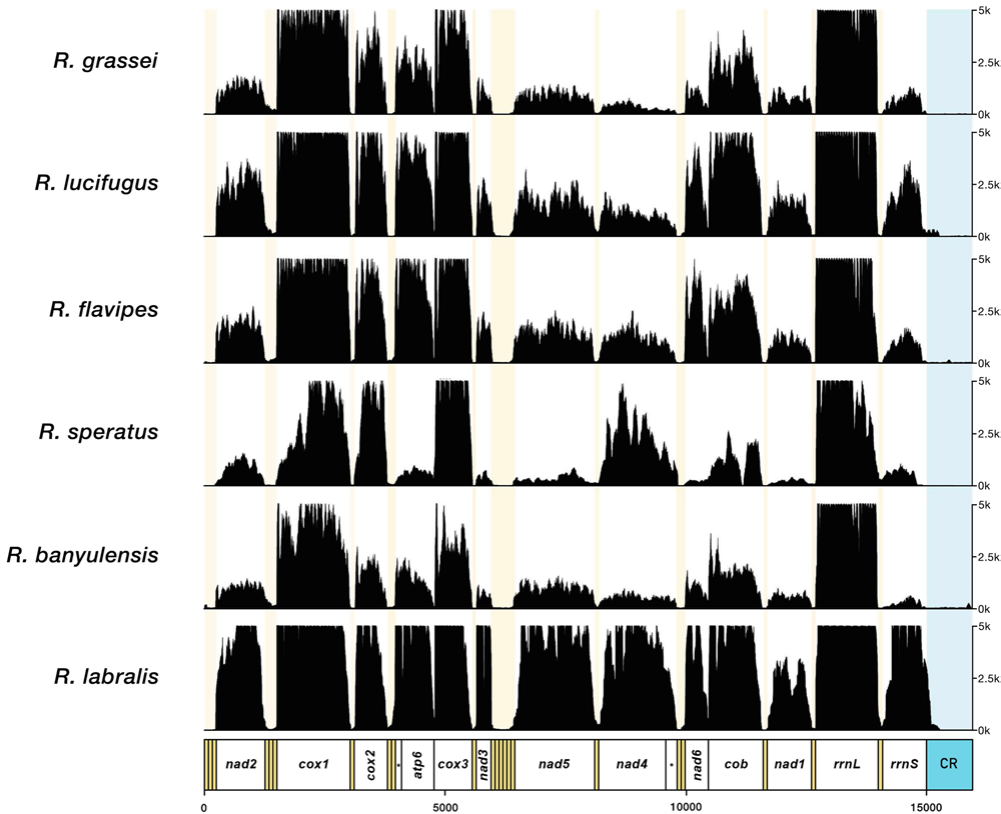
Reads coverage along mitogenomes reconstructed using congeneric references. Coverage has been capped at 5,000 × for graphical purposes; shaded areas indicate tRNAs and the control region.

Sequence coverage was highly variable among genes, but in all cases PCGs and rRNAs were well covered (Fig. 3). tRNA coverage was comparatively much lower than that of the other genes, and this holds for all six *Reticulitermes* species. In particular, manually reconstructed tRNAs showed a drop in the fold coverage below the 4×. This is could be a consequence of their short sequence length: during the size-selection step of the Illumina library preparation protocol it is likely that their mature forms are removed. However, being also part of the non-mature polycistron, we successfully assembled and annotated most of them from RNA-Seq experiments with the automated approach. Overall, the gene coverage profiles obtained (Fig. 3) nicely matche to the gene expression profiles observed in other insects^14,20^ and vertebrate mitogenomes^16,21^, suggesting a similar pattern of differential gene expression also in termites.

Mitogenomes obtained using congeneric and intra-familial initial references were nearly identical: up to seven nucleotide positions show polymorphism over the entire sequence length (Suppl. Table S3). This variation might indicate the presence of heteroplasmy, similarly to what has been previously reported in many organisms^22,23^. Alternatively, mitogenomes sequence identity can be affected by the choice of assembling tools and approaches (de novo or reference-based) used to generate the final assembly^24^. Mitogenomes recovered from RNA-Seq data show divergences ranging from 0.0% to 3.5% with conspecific references (Suppl. Table S4): most of nucleotide substitutions (ranging from 68.9% to 76.9%) occur at the third codon position of protein-coding genes, compatibly with the genetic variability among individuals of the same species (Suppl. Table S5).

When our pipeline was run on references with artificial rearrangements (Supplementary Fig. S3), results do not differ from those obtained using the original references: it can be clearly seen that no chimeric contigs are generated with the different gene order of the references and the correct genes order is obtained in all instances. This can be explained by taking into account the way the pipeline works: mapping on the initial references, and on reconstructed contigs in the subsequent iterations, is only used to recover mitochondrial reads but the assembly is performed de novo at every iteration. Therefore, the gene order resulting at the end of the process (and at the end of every iteration) is given only by the information stored within collected reads.

### Phylogenetic validation of *Reticulitermes* mitogenomes

**Figure 4:**
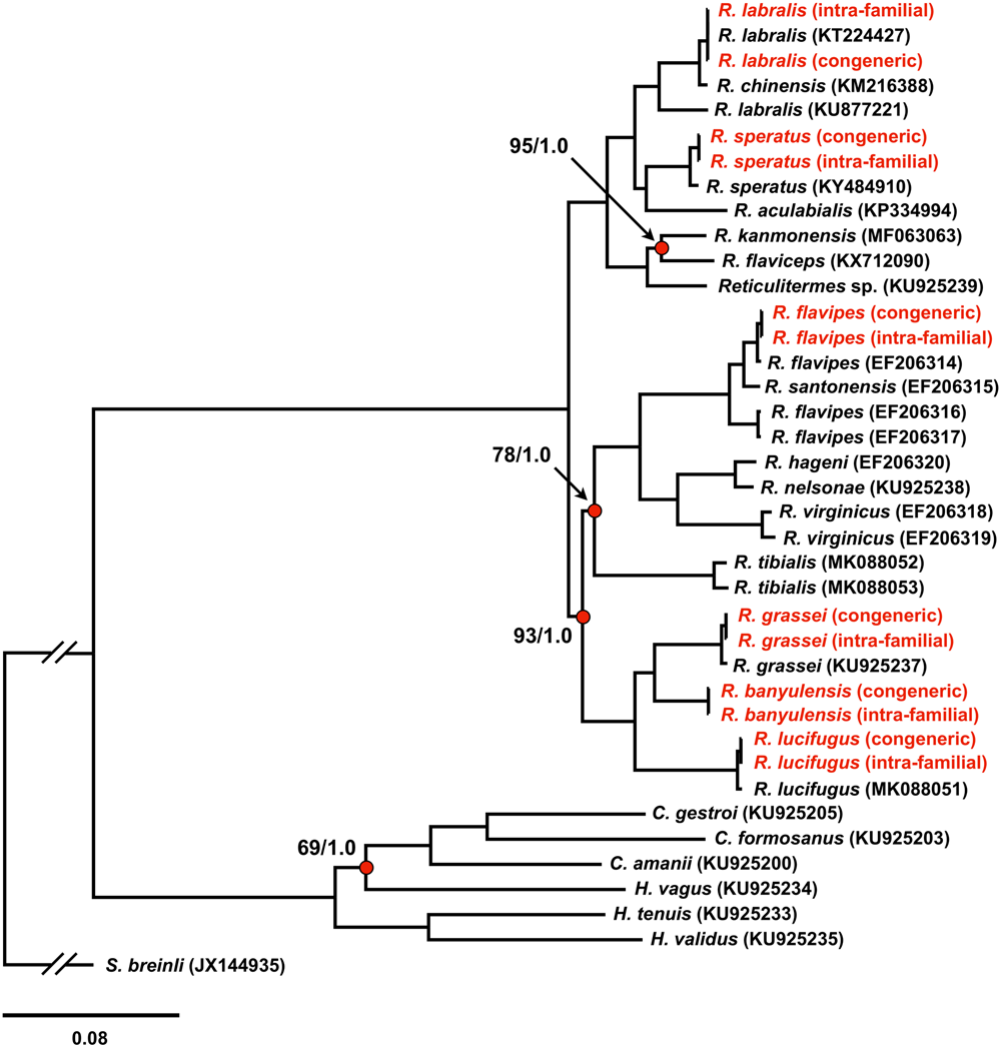
Mitogenomes phylogenetic analysis. Schematic drawing of Maximum Likelihood (−*lnL* = 78672.19) and Bayesian inference (−*lnL* = 78489.80). Maximum bootstrap (=100%) and posterior probability (=1.0) values are omitted, while they are reported on nodes showing supports lower than the maximum (indicated with red dots). Mitogenomes assembled from RNA-Seq are highlighted in red.

In order to assess the accuracy of the mitogenomes obtained from RNA-Seq experiments we tested them in a phylogenetic framework. Our Bayesian and Maximum Likelihood trees (Fig. 4) were congruent with published phylogenetic trees^18,19^. All mitogenomes isolated from RNA-Seq experiments clustered with conspecific DNA-obtained sequences, with maximum node support. Moreover, mitogenomes assembled using different initial references (congeneric or intra-familial) grouped together, with branch lengths equal or close to zero (Fig. 4).

## Conclusions

Although the use of a straight-forward approach, such as the one we applied in this study, appears enough to carry out the task of reconstructing mitochondrial genomes from transcriptome data, some limitations emerged. Some tRNAs and control regions were not reconstructed, but these gaps could be filled by a manual procedure. As evidenced by the coverage analysis, these regions showed a decidedly lower coverage (<4×) with respect to other mitogenome regions, as also observed in other systems^14,16,20,21,25^, and this may have led to fragmented reconstruction. Although we easily overcome the problem by manually assembling gap regions, this may cast some doubts on the scalability of the method when trying to obtain tRNAs and the control region. Most systematics and phylogenetic studies rely on PCGs and rRNA only, but the potential utility of tRNAs should be not overlooked as their inclusion in these analyses may help to improve the inference^1^. Moreover, the reconstruction of gene order and of small non-coding regions can be also important to mark rare genomic changes that could be useful for identifying clades in absence of (or in addition to) the phylogenetic signal of substitutions^26,27^. Finally, information on the control region sequence may be relevant for explaining phylogenetic biases or artefacts obtained in mitochondrial genomes analyses^28^.

Our approach has been tested in the context of insect mitochondrial genomes, which are well known for their mostly conserved structure and the compact size (typically 15–18 kb)^1^. A wide variety of mitochondrial genome size can be found, especially outside metazoan, but some evidences indicated that even mitogenomes rich of non-coding sequences are fully transcribed^12,15^. While this would suggest that it can be possible to recover their complete mitogenome, further experiments are required to evaluate these peculiar conditions.

In this view, possible future development of pipelines running on RNA-Seq data for mitogenome assembly should take into account such challenges. However, we successfully recover the six whole mitogenomes, even in those samples where contaminants and/or random similarities with nuclear transcripts outnumber genuine mitochondrial contigs. In these two instances, namely in *R. speratus* and *R. labralis*, the only issue was a small increase of computational time across the whole process.

Overall, we showed that mitochondrial genome sequences can be accurately reconstructed from transcriptome data using an iterative reference mapping and de novo assembly approach. None of the RNA-Seq data employed in this study were initially generated to investigate mitochondrial aspects^29,30^; the proposed approach has the potential to generate a large number of new mitochondrial genomes for non-model species, by exploiting the increasing amount of publicly available RNA high-throughput sequencing data. This approach can be relevant for molecular taxonomy and systematics but also to investigate various aspects of mitochondrial genome biology^10,31^, such as transcription^32^ and polyadenylation profiles^21^.

## Material and Methods

### Data information

The 12 complete mitochondrial genomes of *Reticulitermes* species sequenced to date have been downloaded from GenBank (accessed on January 2019), alongside the complete mitochondrial genomes of 44 *Coptotermes* and 28 *Heterotermes* (Table 2; Suppl. Table S6). We used the mitogenome of S*chedorhinotermes breinli* (Genbank accession number: JX144935) as outgroup to root phylogenetic trees^18,19,27^.

**Table 2:** Data information for Illumina RNA-Seq experiments and conspecific mitogenomes obtained from NCBI Genbank database.

Species	SRA acc. no.	Gbp	Reads length	Conspecific mitogenomes
*Reticulitermes flavipes*	SRR1325101	2.4	51 (single end)	KY484910
				EF206314
				EF206315^a^
				EF206316
				EF206317
*Reticulitermes grassei*	SRR1325103	1.5	51 (single end)	KU925237
*Reticulitermes banyulensis*	SRR5253660	1.3	51 (single end)	—
*Reticulitermes lucifugus*	SRR1325112	2.3	51 (single end)	MK088051
*Reticulitermes labralis*	SRR5808263	9.1	150 (pair end)	KT224427
				KU877221
*Reticulitermes speratus*	DRR030843	3.4	93 (pair end)	KY484910

^a^This was attributed to *R. santonensis*, which is synonym species of *R. flavipes*.

We selected the RNA-Seq Illumina reads of six species of *Reticulitermes* from which we attempted to extract mitogenomes (Table 2). For five species (*R. flavipes*, *R. grassei*, *R. lucifugus*, *R. labralis* and *R. speratus*), a conspecific mitochondrial genome sequence was already available, which we used to assess the accuracy and completeness of our assemblies. For *R. banyulensis*, no mitogenome reference was available, and we here present the first complete mitochondrial genome.

### Mitochondrial genome reconstruction pipeline

**Figure 5:**
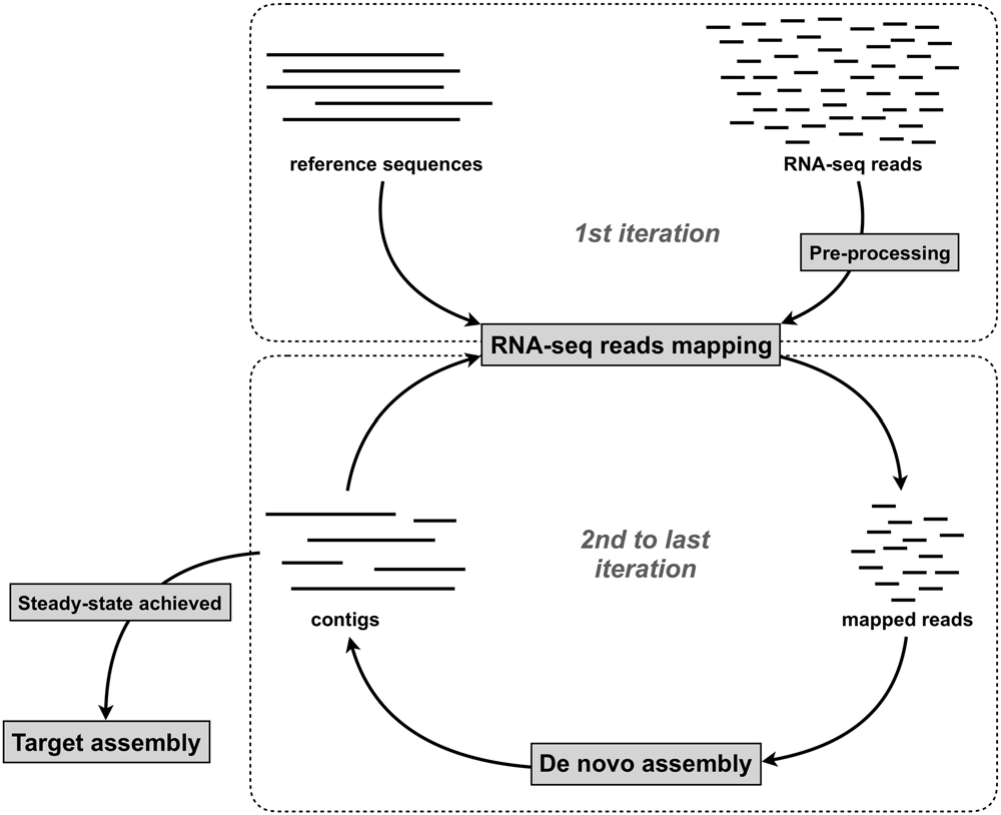
Conceptual map of the pipeline implemented for mitogenome reconstruction from RNA-Seq raw reads.

Raw reads were downloaded with Fastq-dump, quality-checked with FastQC^33^ and trimmed and clipped with Trimmomatic^34^, using parameters ILLUMINACLIP:TruSeq3-PE.fa:1:30:10 LEADING:3 TRAILING:3 SLIDINGWINDOW:25:33 MINLEN:45 for reads of over 50 bp length and parameters ILLUMINACLIP:TruSeq3-PE.fa:1:30:10 LEADING:3 TRAILING:3 SLIDINGWINDOW:25:33 MINLEN:90 for reads of over 90 bp length. In order to check the assembly process also during intermediate steps, we set up an iteration-based pipeline (available at https://github.com/mozoo/mitoRNA), summarized as follow (Fig. 5):

(i) in the first step, all transcriptome reads are mapped to reference mitochondrial genomes with Bowtie2^35^ in local mode with parameters set to–very-sensitive-local, whose default settings are -D 20 -R 3 -N 0 -L 20 -i S,1,0.50, but allowing one mismatch in the seed alignment (−N 1) and by reducing the length of the seed substrings to align (−L 10). This increases the sensitivity of the mapping.
(ii) in the second step, mapped reads are de novo assembled by Trinity^36^ with the settings* --no_normalize_reads --min_contig_length* 150 bp, which remove the in silico reads normalization step and set the minimum length for a contig to be assembled at 150 bp.

Generated contigs are then used as a new reference for successive iterations. In order to test whether reference mitogenomes affect the reconstruction of the RNA-Seq-derived mitogenomes, we excluded conspecific sequences from the initial reference list used in the first step of the first iteration (*i.e*. when analyzing the RNA-Seq reads of a given species, the mitogenome(s) of the same species was excluded from the list of reference genomes). In addition, to evaluate the effect of phylogenetic relatedness of the starting reference mitogenomes, we used either *Reticulitermes* mitogenomes (congeneric references) or *Coptotermes* + *Heterotermes* mitogenomes (intra-familial) during the first step of the first iteration.

For each transcriptome analyzed, we evaluated the progress of this iterative approach using both congeneric and intra-familial starting references, stopping the process after 10 iterations. At the 10th iteration, then, we recovered a variable number of contigs which were validated and scaffolded with *blastn*^37^ using default parameters against the closest, non-conspecific reference mitogenome. We then merged the scaffolded contigs using Aliview^38^. Where necessary (see Results) gaps in reconstructed molecule were filled by BLASTing transcriptome reads against the homolog portion of the reference mitochondrial genomes, then reads were assembled with CAP3^39^ and aligned to the scaffold using MAFFT v.7^40^.

In order to assess the impact of using references showing a different gene order with respect to the target mitogenome, we generated artificial rearrangements of the congeneric references (Supplementary Fig. S3) and we then re-run our pipeline using the rearranged molecules as initial references.

We annotated the 22 tRNA genes, the 13 protein coding genes (PCGs), and the two ribosomal RNA genes manually, aided by congeneric published reference sequences.

### Quality assessment and phylogenetic analysis

During the mapping process, it is possible that some reads derived from either contaminant (if any) or nuclear DNA can leak into the iterative process, because of random homology, and assembled into contigs. Therefore, contigs generated at the 10th iteration were analyzed to check if they derive from contaminant reads (contaminants leakage) or from nuclear reads (nuclear leakage). To estimate the contaminants leakage, we identified contigs that did not match with insects using DIAMOND^41^. Then, to estimate the nuclear leakage, we identified contigs matching to insects but not to the reconstructed mitochondrial genomes by means of a *blastn* search.

The coverage of all newly reconstructed mitogenomes was determined by mapping reads with Bowtie2 and analysing the output with SAMtools^42^. Newly obtained mitogenomes were annotated based on homologies with previously published ones, and all protein coding genes (PCGs) were manually inspected for open reading frame correctness. We also estimated tRNAs secondary structure for both DNA-derived and RNA-derived mitogenomes using Mitos2^43^ (available at: http://mitos2.bioinf.uni-leipzig.de/index.py).

Phylogenetic and nucleotide divergence analyses have been carried out using PCGs and rRNAs. Each gene was aligned separately using MAFFT with the option* --auto* for protein coding gene (PGC) and with the option* --X-INS-i* for the two rRNA genes. We omitted control regions from the final matrix as they were only partially assembled. After concatenation, the final matrix included 37 sequences spanning 13,547 nucleotide positions. Phylogenetic trees were reconstructed using Bayesian inference and Maximum Likelihood approaches. Model selection and phylogenetic inference were carried out through CIPRES Science Gateway (www.phylo.org)^44^. For both Bayesian and Maximum Likelihood approaches, the best-fit models of nucleotide substitution were identified using IQ-TREE Model Selection^45^, using the edge-linked parameter and the TESTNEWMERGE flag (Suppl. Tables S7 and S8). The ML search was run with 1,000 ultrafast bootstraps replicates using IQ-TREE v1.6.1^46^. The Bayesian inference was carried out using MrBayes v. 3.2.6^47^: two Markov chains Monte Carlo (MCMC) were run simultaneously for 10,000,000 generations and were sampled every 1,000 generations. Burn-in was set at a conservative threshold of 25%. Average deviation of split frequencies fell below 0.01 within 1 million generations, indicating the chain reached convergence.

## Supplementary information

Supplementary Information

## Data Availability

Mitogenomes assembled from RNA-Seq experiments are available on Fig Share under the 10.6084/m9.figshare.7181969.
