# Supplementary material for: Complete mitochondrial genomes from transcriptomes: assessing pros and cons of data mining for assembling new mitogenomes
Source: Sci Rep. 2019 Oct 15;9:14806. doi: 10.1038/s41598-019-51313-7 (PMC6794255; doi:10.1038/s41598-019-51313-7)

Supplementary Information

Complete mitochondrial genomes from transcriptomes: assessing pros and cons of data mining for assembling new mitogenomes.

Giobbe Forni, Guglielmo Puccio, Thomas Bourguignon, Theodore Evans, Barbara Mantovani, Omar Rota-Stabelli, Andrea Luchetti

Table S1. Contig filtering for contaminants, based on NCBI *taxid* (indicated in parentheses).

	***R. labralis* congeneric**	***R. labralis* intra-familial**	***R. speratus* congeneric**	***R. speratus* intra-familial**
Total contigs	34439	33906	262	157
Total hits with e-value ≤ 0.001	11285	11013	157	95
No hits	67.23%	67.52%	40.08%	39.49%
Bacteria (2)	74	83	0	0
Viridiplantae (33090)	161	123	0	0
Fungi (4751)	89	125	0	0
Vertebrata (7742)	216	192	4	4
Hexapoda (6960) - excluding termites (1912919)	880	830	22	8
Insecta (50557)	9427	9264	134	75
Reticulitermes (36988)	187	210	36	20
Contaminants or unassigned	72.63%	72.68%	48.85%	52.23%

Table S2. Control regions (CR) length in reference and RNA-Seq derived mitogenomes (using congeneric or intra-familial reference in the first step of the first iteration).

Species	Genbank acc. nos.	CR length (bp)	CR RNA-Seq from congeneric ref. (bp)	CR RNA-Seq from intra-familial ref. (bp)
Reticulitermes flavipes	KY484910	1099	1194	107
	EF206314	1751		
	EF206315	1751		
	EF206316	1751		
	EF206317	1751		
Reticulitermes grassei	KU925237	14	926	n\a
Reticulitermes banyulensis	-	n\a	1024	n\a
Reticulitermes lucifugus	MK088051	1471	1340	293
Reticulitermes labralis	KT224427	1308	1127	736
	KU877221	1098		
Reticulitermes speratus	KY484910	1098	955	113

Table S3. Nucleotide substitutions between RNA-Seq derived mitogenomes obtained with either congeneric or intrafamilial starting references.

Species	Number of differences[a]	Position (nt)	Gene	Strand	Congeneric variant	Intra-familial variant	Outcome congeneric	Outcome intra-familial	Coverage congeneric	Coverage intra-familial
Reticulitermes flavipes	0	-	-		-	-	-	-	-	-
Reticulitermes lucifugus	4	9722	ND4L	-	G	A	AGC (s)	AGT (s)	713	732
		9800			A	T	TTT (f)	TTA (l)	50	61
		9803			A	C	TGT (c)	TGG (w)	42	52
		3031	tRNA-Leu	+	T	C			421	148
Reticulitermes grassei	4	14825	12S	+	A	T			1366	1331
		388	ND2	+	A	G	ACA (t)	ACG (t)	1617	1559
		548			G	A	GGG (g)	GGA (g)	1859	1616
		6307	tRNA-Glu	+	A	C			8	7
Reticulitermes banyulensis	4	14793	12S	+	A	C			399	661
		14795			C	A			337	640
		14796			T	C			318	633
		14797			C	T			304	619
Reticulitermes speratus	7	14576	12S	+	A	T			1087	1052
		14579			-	A			-	1031
		14580			-	A			-	1033
		14272			-	C			-	1004
		6479	ND5	-	A	C	TTT (f)	TTG (l)	635	998
		6538			G	C	CAT (h)	GAT (f)	359	836
		6241	tRNA-Ser	+	C	G			8	11
Reticulitermes labralis	6(4)	14246	12S	+	T	-			182	-
		14247			C	A			3701	3827
		11580	beteween CYTB and tRNA-Ser2		C	G			34	24

[a] differences excluding CR in parentheses

Table S4. Nucleotide substitutions (% divergence) between conspecific reference sequences drawn from Genbank versus RNA-Seq derived mitogenomes obtained with either congeneric or intrafamilial references in the first step of the first iteration.

**Species**	**Conspecific reference**	***vs* RNA Seq (congeneric)**	***vs* RNA Seq (intrafamilial)**
Reticulitermes flavipes	EF206314	46 (0.3%)	47 (0.3%)
	EF206315 [a]	208 (1.3%)	189 (1.3%)
	EF206316	325 (2.2%)	326 (2.2%)
	EF206317	323 (2.2%)	324 (2.2%)
	KU925236 [b]	248 (2.5%)	248 (2.5%)
Reticulitermes grassei	KU925237	69 (0.5%)	72 (0.5%)
Reticulitermes lucifugus	MK088051	58 (0.4%)	55 (0.4%)
Reticulitermes labralis	KT224427	3 (0.0%)	3 (0.0%)
	KU877221	511 (3.5%)	511 (3.5%)
Reticulitermes speratus	KY484910	115 (0.8%)	112 (0.8%)

[a] *R. santonensis* is synonym species of *R. flavipes*

[b] incomplete sequence

Table S5. Number of nucleotide substitutions in protein-coding genes between RNA-Seq derived mitogenomes obtained with either congeneric or intra-familial starting references and the closest mitogenome obtained by DNA-sequencing.

	***vs* Closest reference**	**1st**	**2nd**	**3rd**
PCGs - all *Reticulitermes*		1059.4	497.7	2985.9
R. flavipes (congeneric)	EF206314	5	4	30
R. flavipes (intra-familial)	EF206314	6	4	30
R. grassei (congeneric)	KU925237	11	4	43
R. grassei (intra-familial)	KU925237	11	4	45
R. lucifugus (congeneric)	MK088051	9	5	34
R. lucifugus (intra-familial)	MK088051	9	5	31
R. labralis (congeneric)	KT224427	0	0	0
R. labralis (intra-familial)	KT224427	0	0	0
R. speratus (congeneric)	KY484910	16	9	63
R. speratus (intra-familial)	KY484910	15	9	62

Table S6. Mitochondrial genomes used as reference (those used for the phylogenetic analysis are marked with an asterisk).

**Species**	**Genbank accession numbers**
Coptotermes acinaciformis	KU925196
Coptotermes acinaciformis	KU925197
Coptotermes acinaciformis	KU925198
Coptotermes acinaciformis	KU925199
Coptotermes amanii	KU925200*
Coptotermes elisae	KU925201
Coptotermes formosanus	KU925203*
Coptotermes formosanus	AB626145
Coptotermes frenchi	KU925204
Coptotermes gestroi	KU925205*
Coptotermes heimi	KU925206
Coptotermes heimi	KU925207
Coptotermes heimi	KU925208
Coptotermes kalshoveni	KU925209
Coptotermes kalshoveni	KU925210
Coptotermes lacteus	KU925211
Coptotermes lacteus	JX144934
Coptotermes michaelseni	KU925212
Coptotermes sepangensis	KU925215
Coptotermes sjoestedti	KU925216
Coptotermes sjoestedti	KU925217
Coptotermes testaceus	KU925218
Coptotermes testaceus	KU925219
Coptotermes testaceus	KR872938
Coptotermes travians	KU925221
Coptotermes travians	KU925222
Heterotermes cf. occiduus	KU925229
Heterotermes cf. occiduus	KU925230
Heterotermes cf. paradoxus	KU925223
Heterotermes cf. paradoxus	KU925224
Heterotermes cf. paradoxus	KU925225
Heterotermes crinitus	KU925226
Heterotermes malabaricus	KU925227
Heterotermes nr. tenuis	KU925228
Heterotermes platycephalus	KU925231
Heterotermes tenuior	KU925232
Heterotermes tenuis	KU925233*
Heterotermes vagus	KU925234*

Heterotermes validus	KU925235*
Reticulitermes aculabilis	KP334994*
Reticulitermes chinensis	KM216388*
Reticulitermes flaviceps	KX712090*
Reticulitermes flavipes	EF206316*
Reticulitermes flavipes	EF206317*
Reticulitermes flavipes	EF206314*
Reticulitermes grassei	KU925237*
Reticulitermes hageni	EF206320*
Reticulitermes kanmonensis	MF063063*
Reticulitermes labralis	KU877221*
Reticulitermes labralis	KT224427*
Reticulitermes lucifugus	MK088051*
Reticulitermes nelsonae	KU92523*
Reticulitermes santonensis	EF206315[a]*
Reticulitermes sp.	KU925239*
Reticulitermes speratus	KY484910*
Reticulitermes tibialis	MK088052*
Reticulitermes tibialis	MK088053*
Reticulitermes virginicus	EF206318*
Reticulitermes virginicus	EF206319*

[a] *R. santonensis* is synonym species of *R. flavipes*

Table S7. Best partition scheme and substitution models for the Maximum Likelihood search in IQ-TREE.

Partition	Subset	Best Model
1	rrnS+rrnL	GTR+F+I+G4
2	atp6_1st+cox2_1st+cox3_1st	TIM+F+G4
3	atp6_2nd+nad1_2nd+nad4_2nd+nad4L_2nd+nad5_2nd	TIM3+F+I+G4
4	atp6_3rd+atp8_3rd+cox2_3rd+cox3_3rd+cob_3rd+nad3_3rd+nad6_3rd	HKY+F+I+G4
5	atp8_1st+atp8_2nd+cob_1st+nad2_1st+nad3_1st+nad6_1st	TIM2+F+I+G4
6	cox1st	TIM2e+G4
7	cox1nd	HKY+F+I
8	cox1_3rd+nad2_3rd	TVM+F+G4
9	cox2_2nd+cox3_2nd+cob_2nd+nad2_2nd+nad3_2nd+nad6_2nd	TPM3+F+I+G4
10	nad1_1st+nad4_1st+nad4L_1st+nad5_1st	HKY+F+G4
11	nad1_3rd+nad4_3rd+nad4L_3rd+nad5_3rd	GTR+F+I+G4

Table S8. Best partition scheme and substitution models for the Bayesian Inference in MrBayes.

Partition	Subset	Best Model
1	rrnS+rrnL	GTR+F+I+G4
2	atp6_1st+cox1_1st+cox2_1st+cox3_1st	GTR+F+G4
3	atp6_2nd+nad1_2nd+nad4_2nd+nad4L_2nd+nad5_2nd	GTR+F+I+G4
4	atp6_3rd+atp8_3rd+cox2_3rd+cox3_3rd+cob_3rd+nad3_3rd+nad6_3rd	HKY+F+I+G4
5	atp8_1st+atp8_2nd+nad6_1st	HKY+F+G4
6	cox1nd	HKY+F+I
7	cox1_3rd+nad2_3rd	GTR+F+G4
8	cox2_2nd+cox3_2nd+cob_2nd+nad2_2nd+nad3_2nd+nad6_2nd	HKY+F+I+G4
9	cob_1st+nad2_1st+nad3_1st	HKY+F+I+G4
10	nad1_1st+nad4_1st+nad4L_1st+nad5_1st	HKY+F+G4
11	nad1_3rd+nad4_3rd+nad4L_3rd+nad5_3rd	GTR+F+I+G4

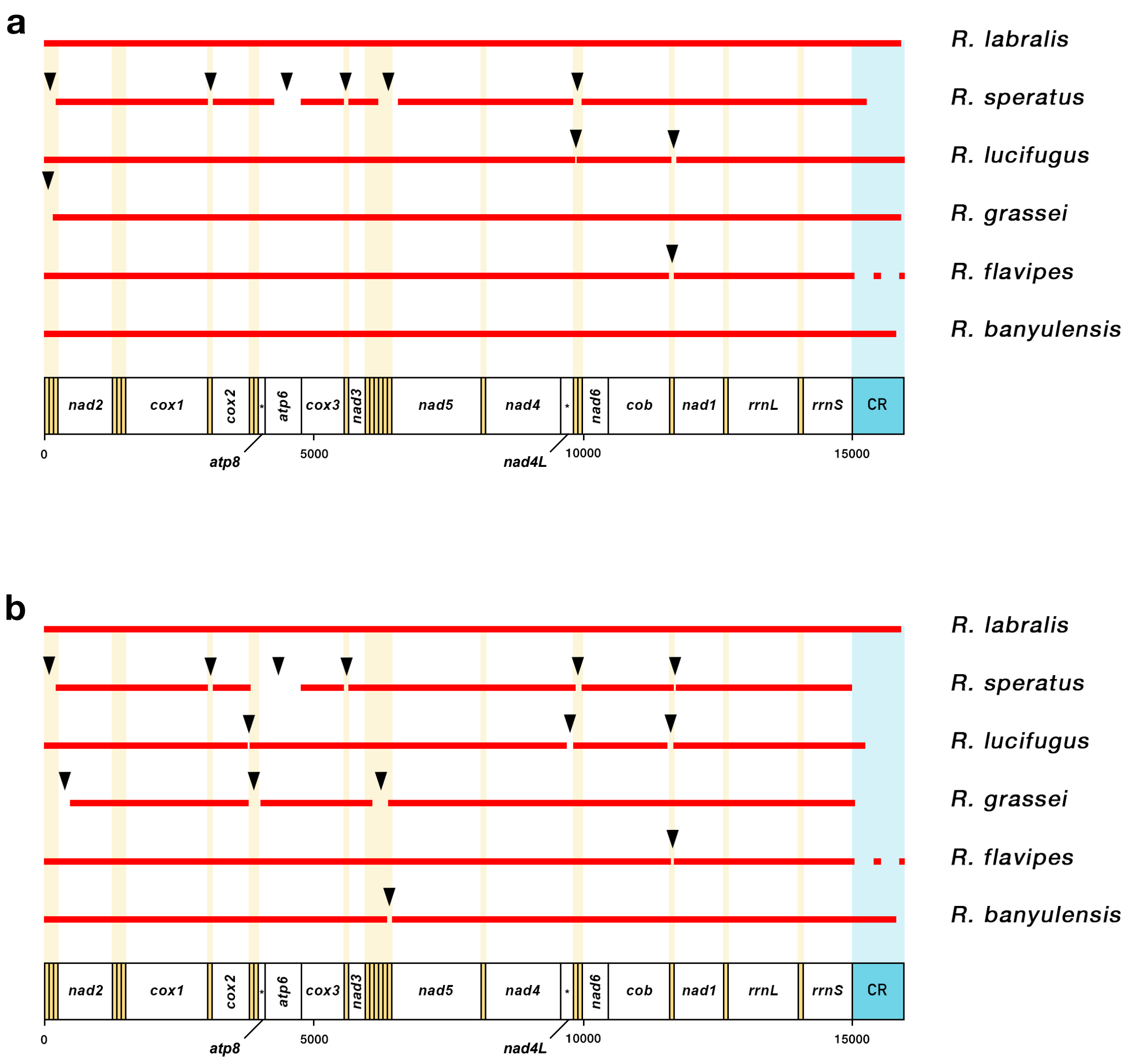

Figure S1. Localization of gap regions, after the 10th iteration, within reconstructed mitogenomes using a) congeneric and b) intra-familial references

Figure S2. tRNAs secondary structure of reconstructed and reference mitogenomes

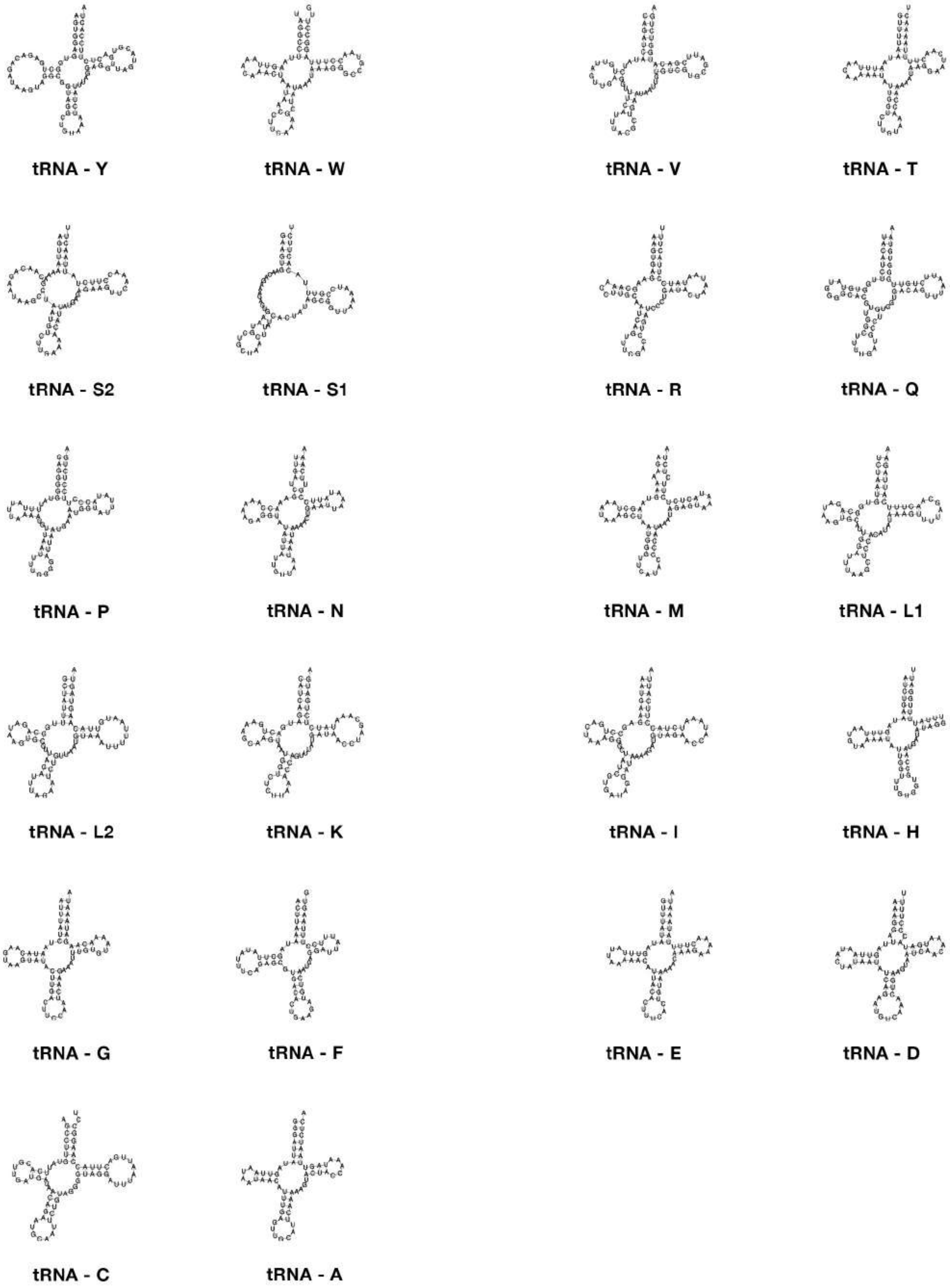

Reticulitermes banyulensis - intrafamiliar starting references

Figure S2. Continued

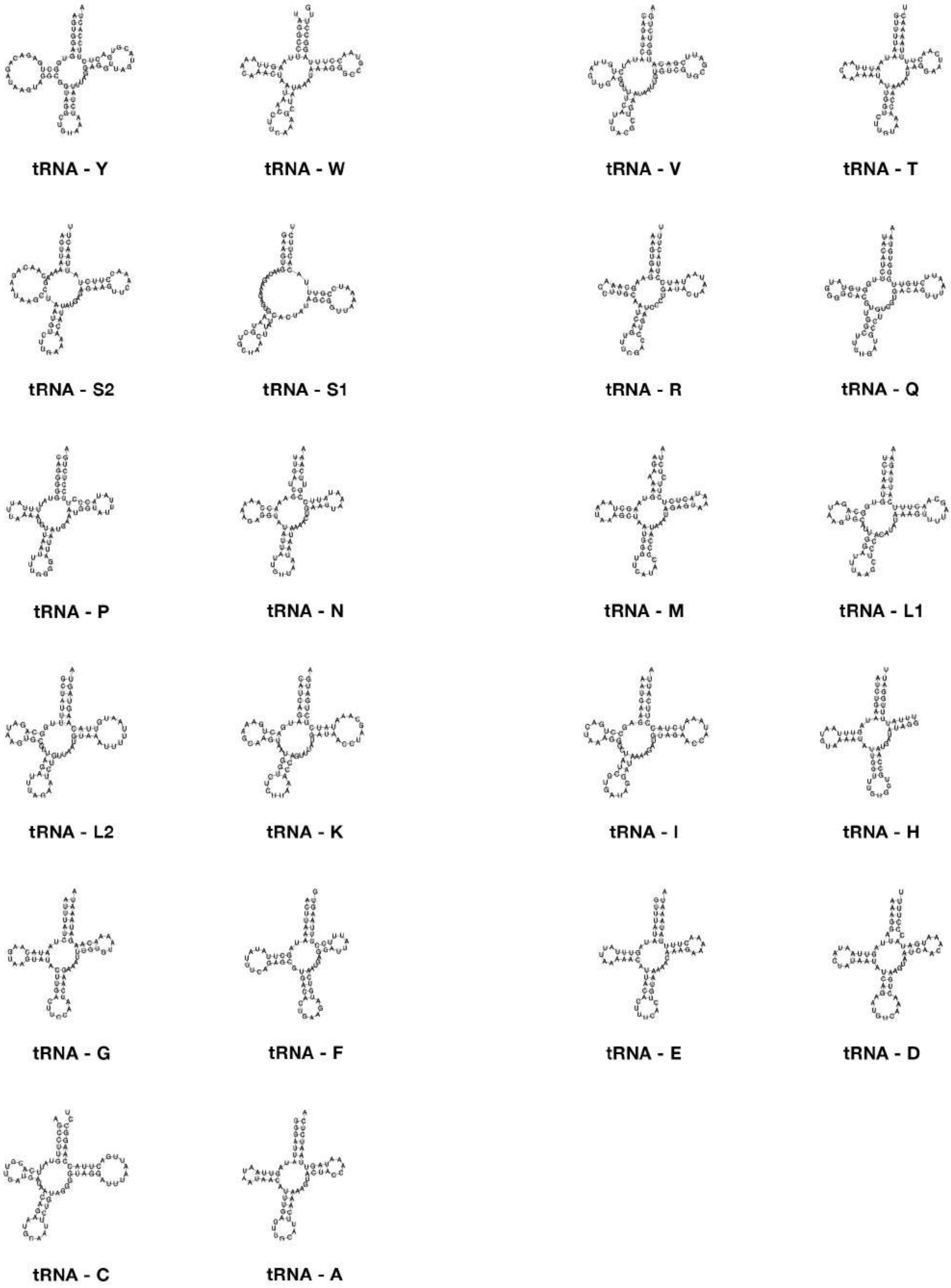

Reticulitermes banyulensis - congeneric starting references

Figure S2. Continued

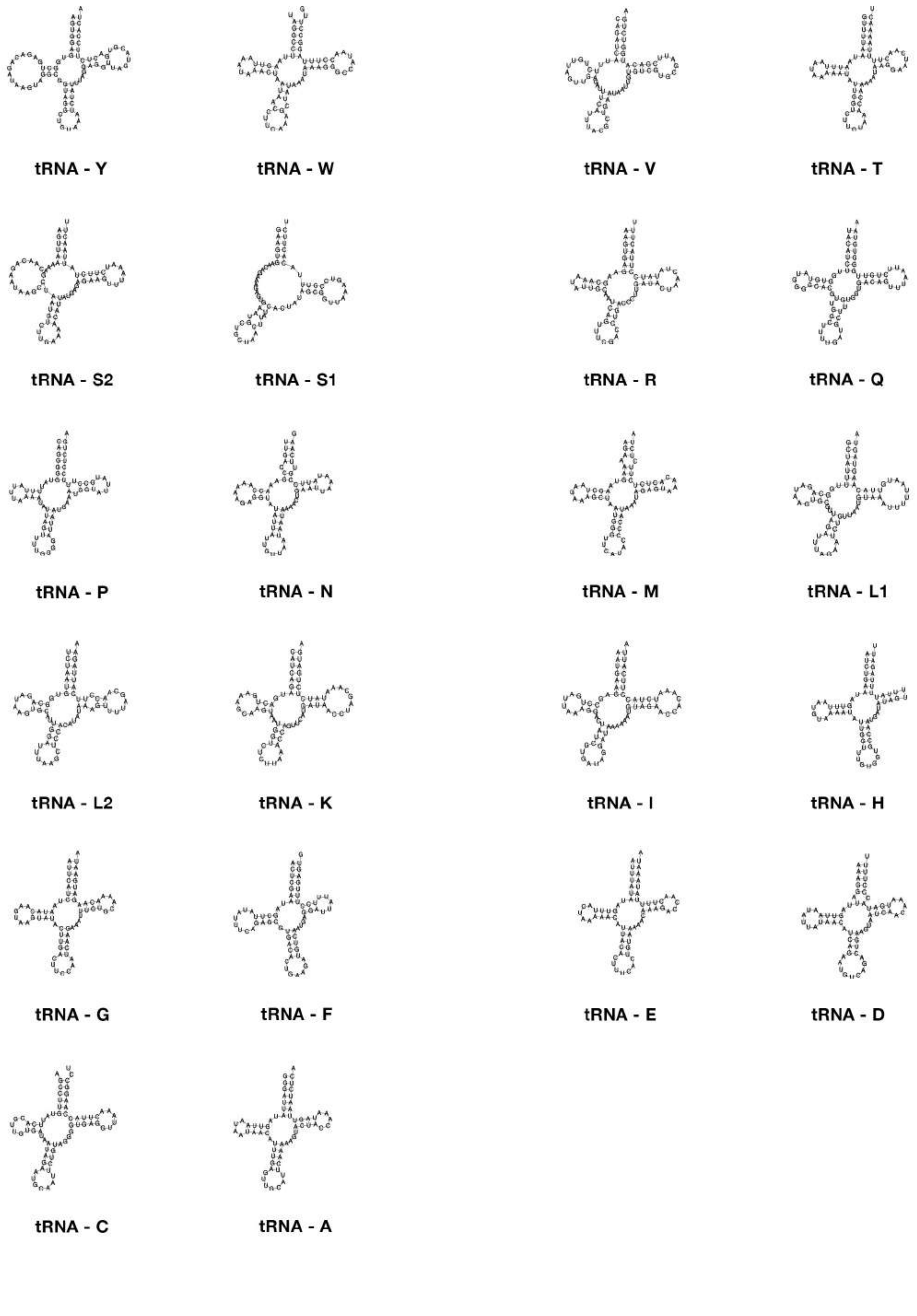

Reticulitermes flavipes EF206314.1

Figure S2. Continued

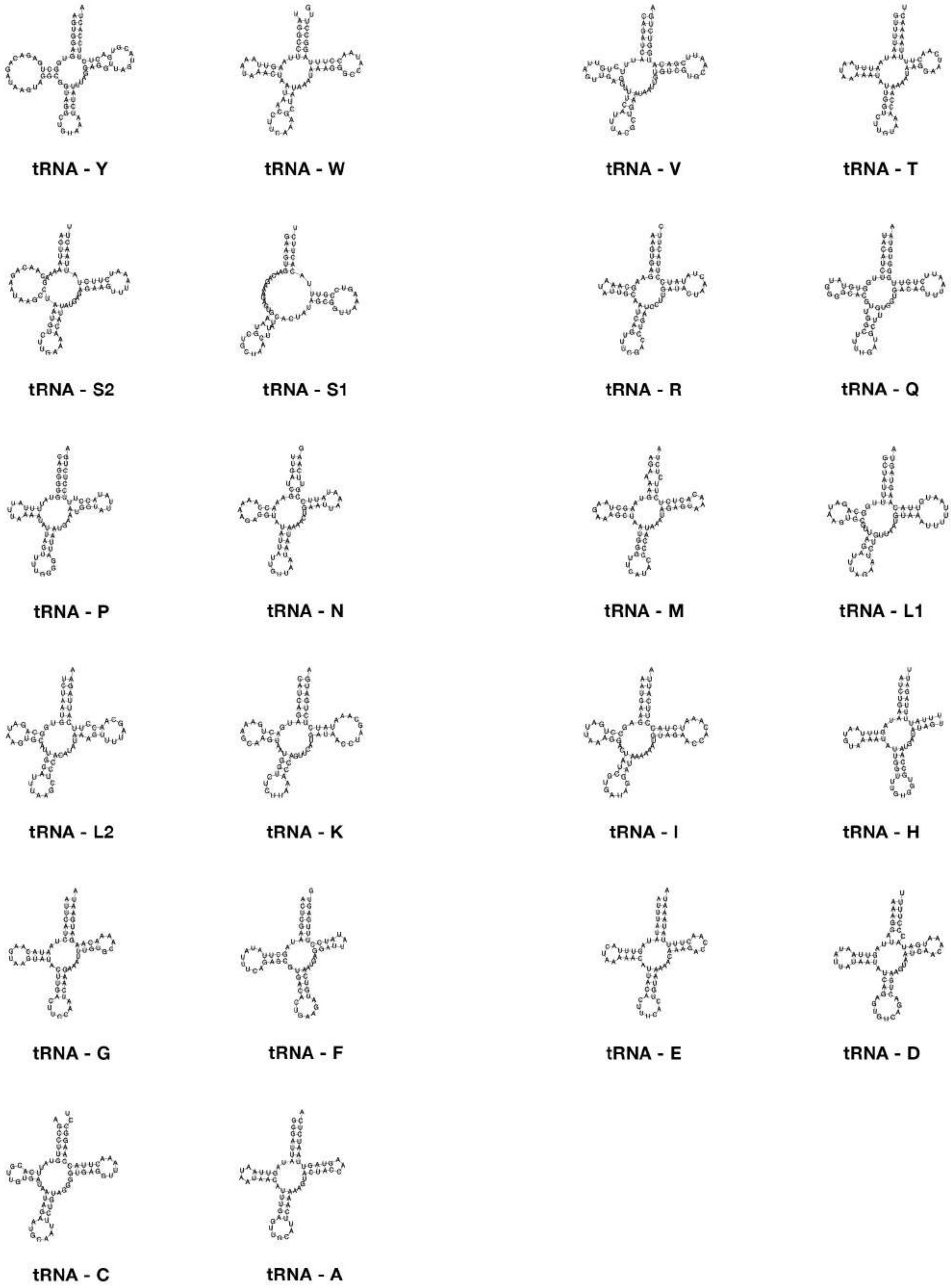

Reticulitermes flavipes - intrafamiliar starting references

Figure S2. Continued

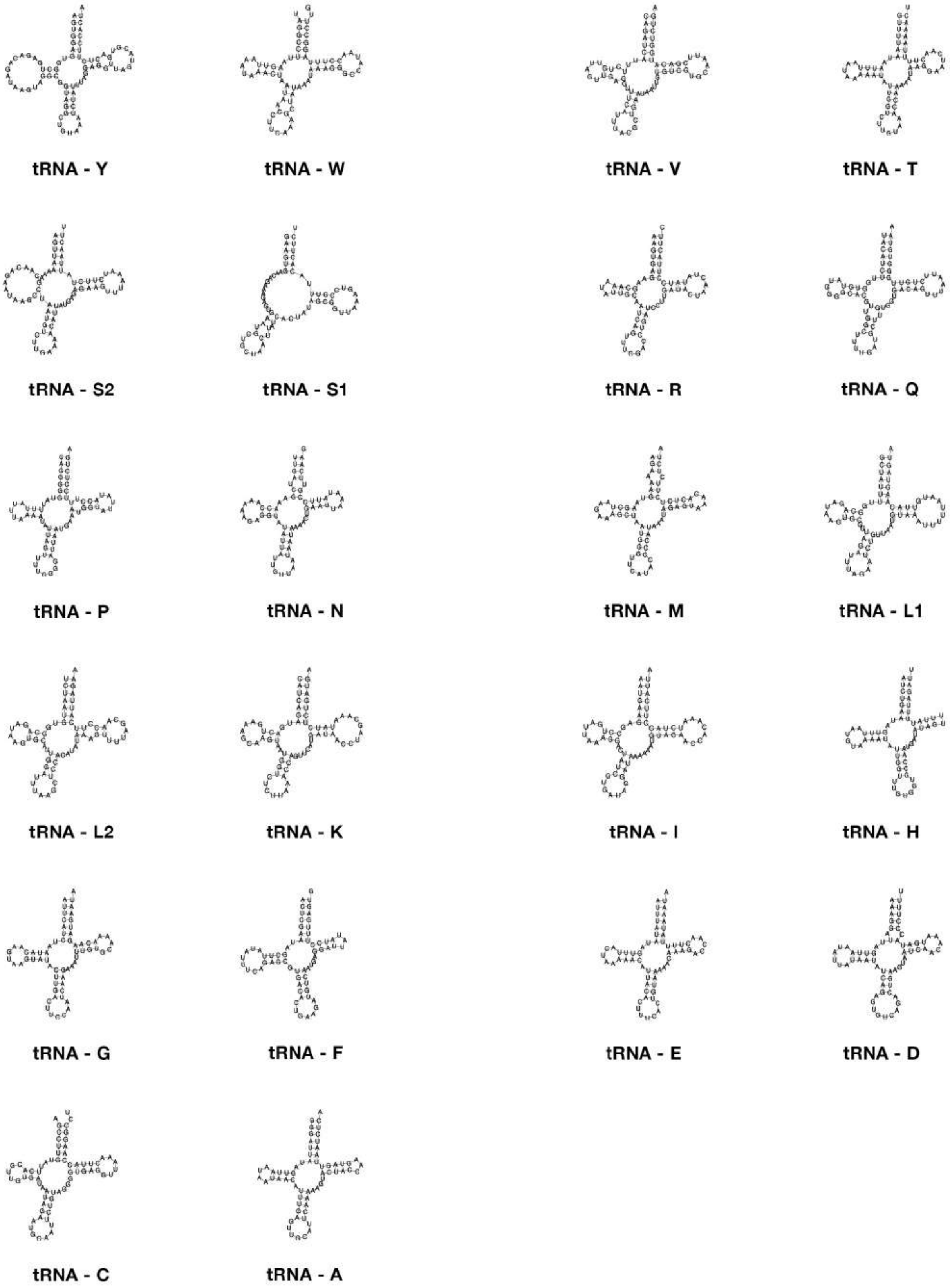

Reticulitermes flavipes - congeneric starting references

Figure S2. Continued

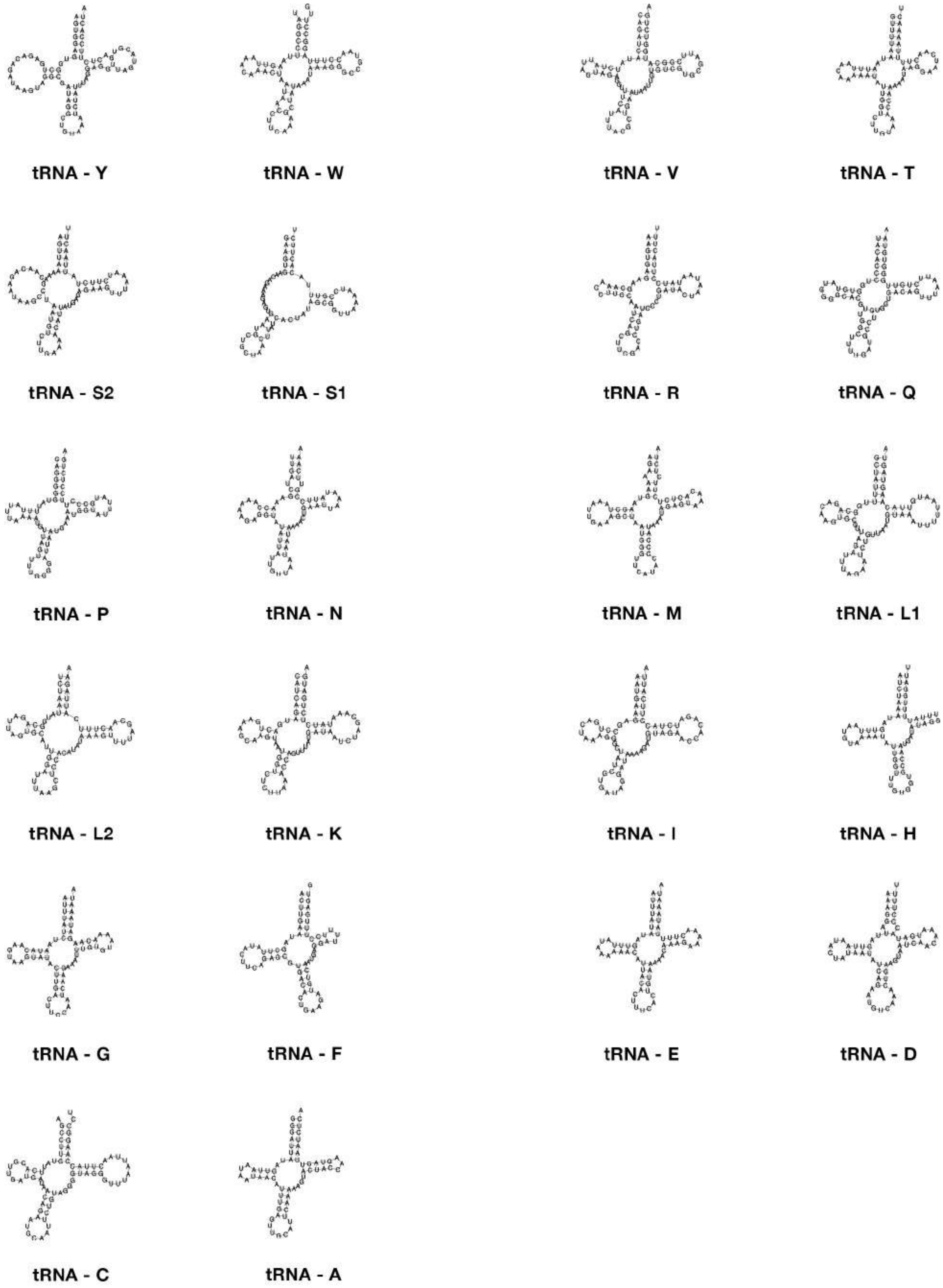

Reticulitermes grassei KU925237.1

Figure S2. Continued

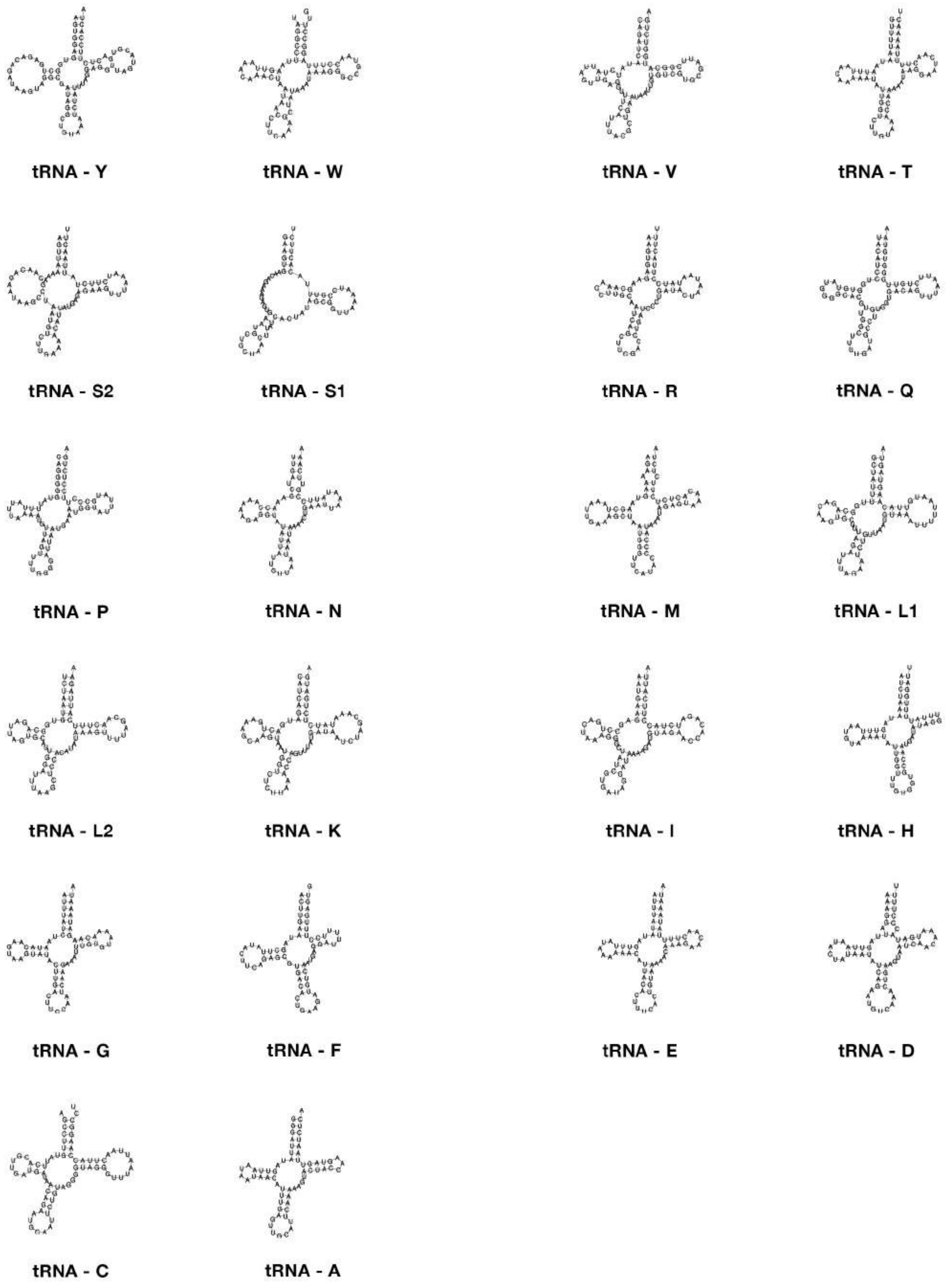

Reticulitermes grassei - intrafamiliar starting references

Figure S2. Continued

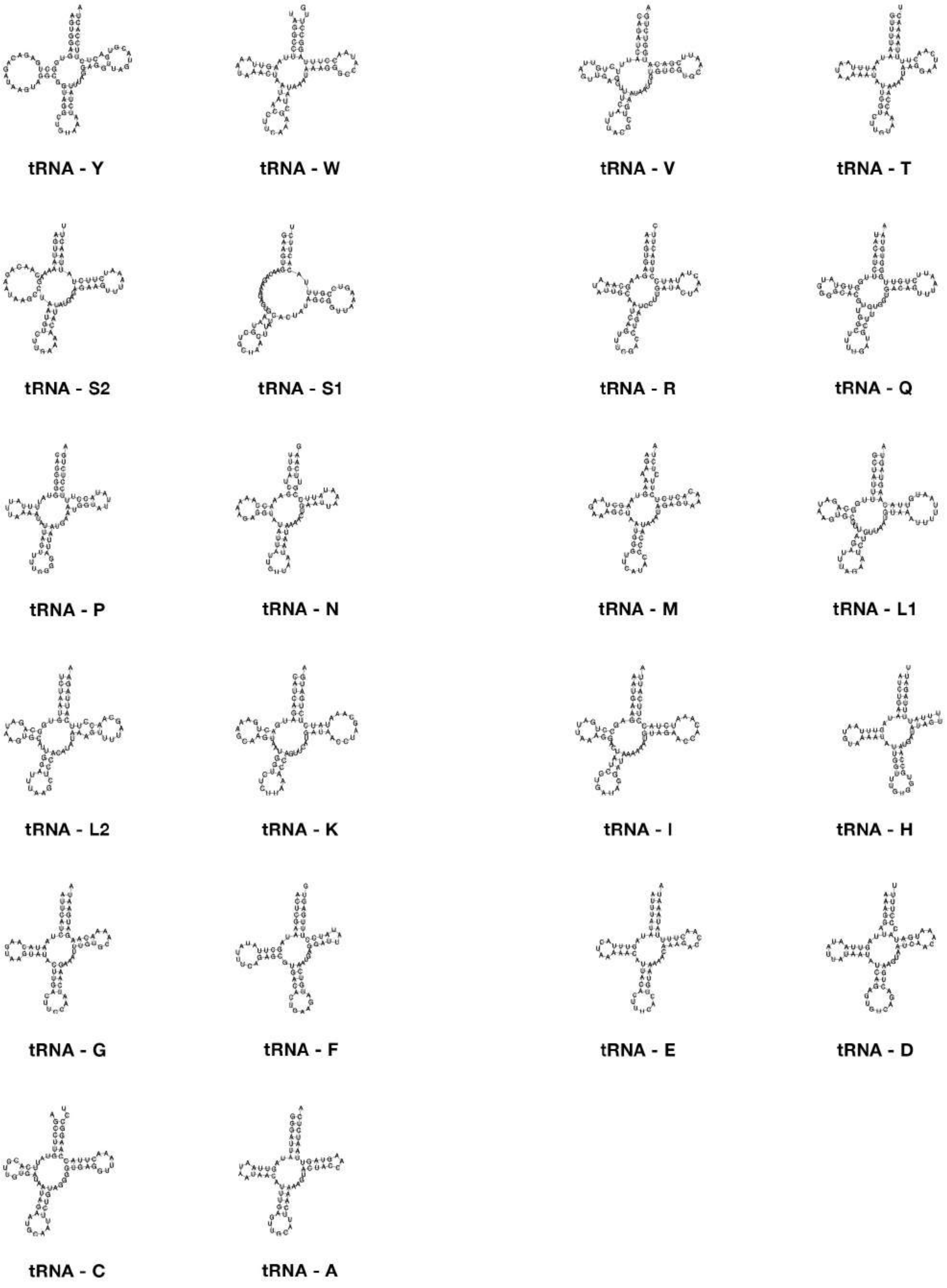

Reticulitermes grassei - congeneric starting references

Figure S2. Continued

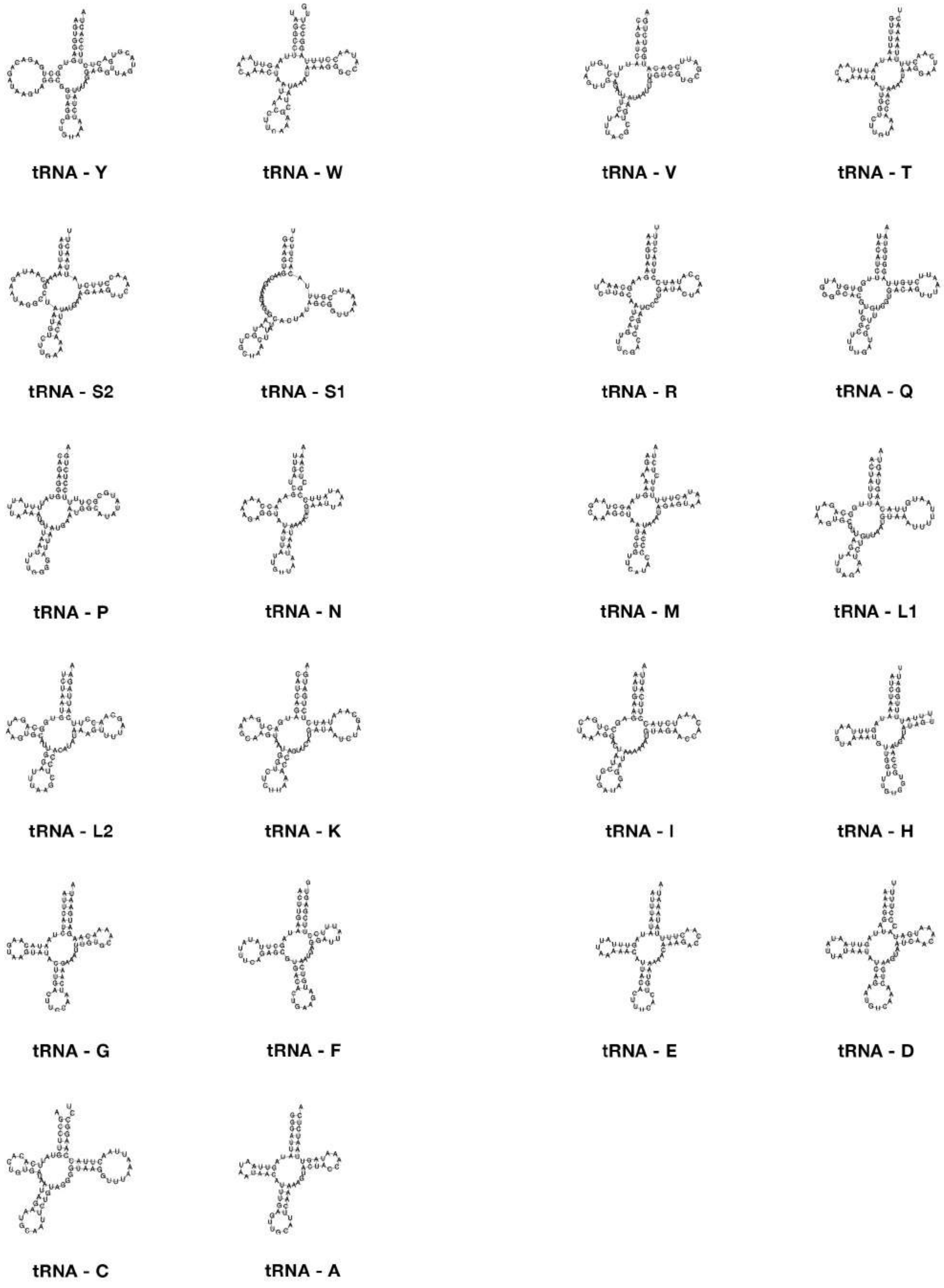

Reticulitermes labralis KT224427.1

Figure S2. Continued

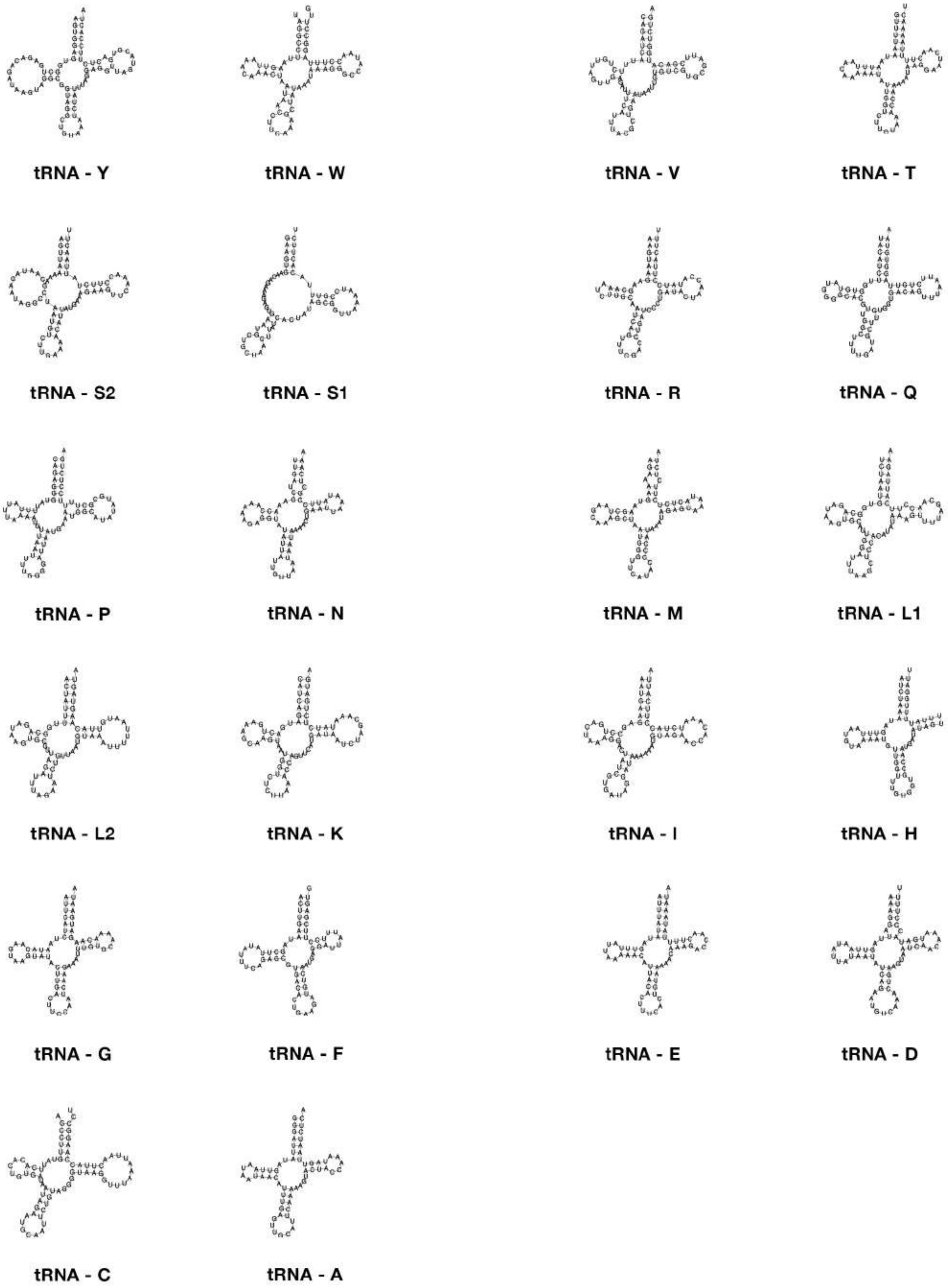

Reticulitermes labralis - intrafamiliar starting references

Figure S2. Continued

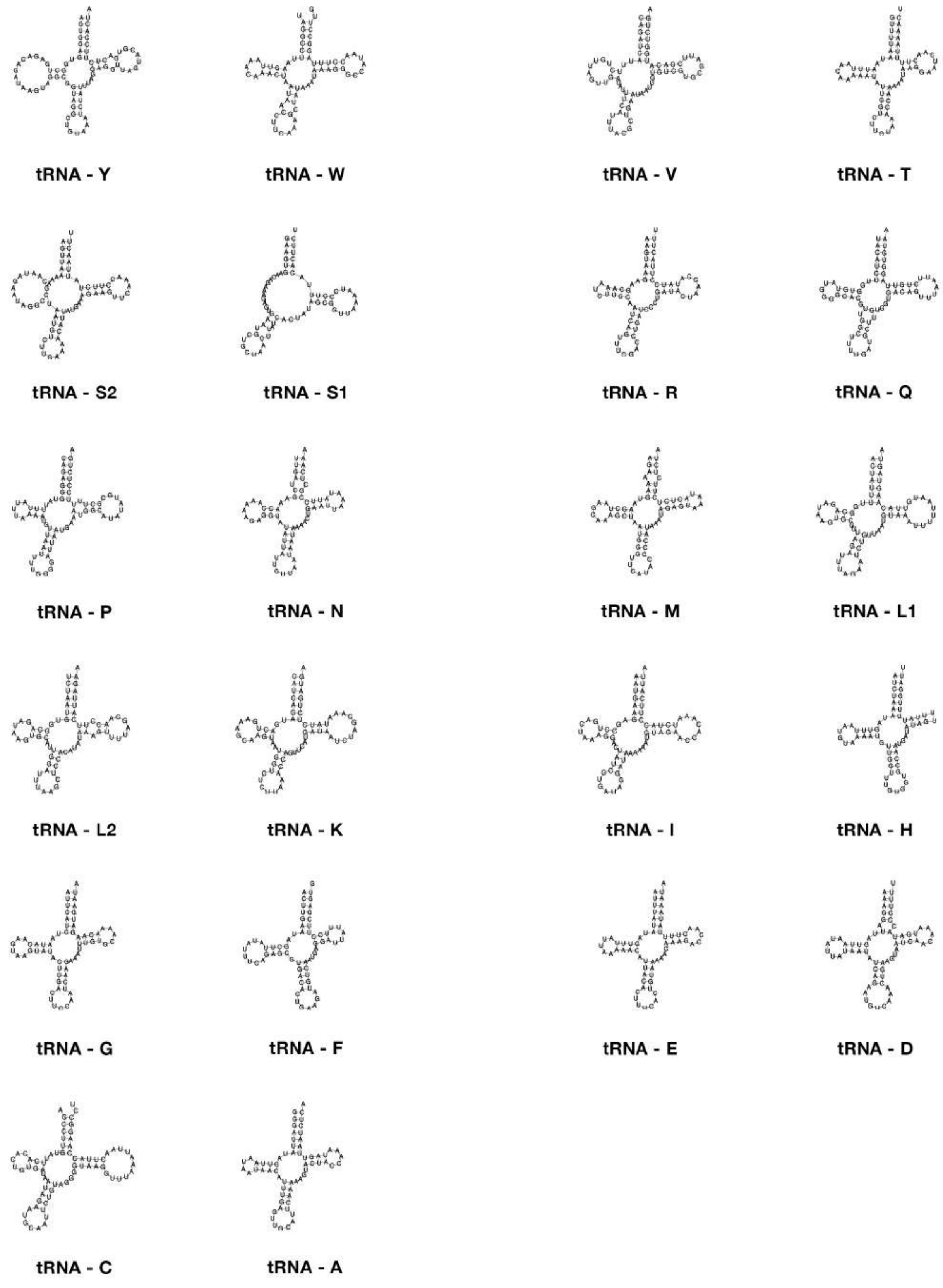

Reticulitermes labralis - congeneric starting references

Figure S2. Continued

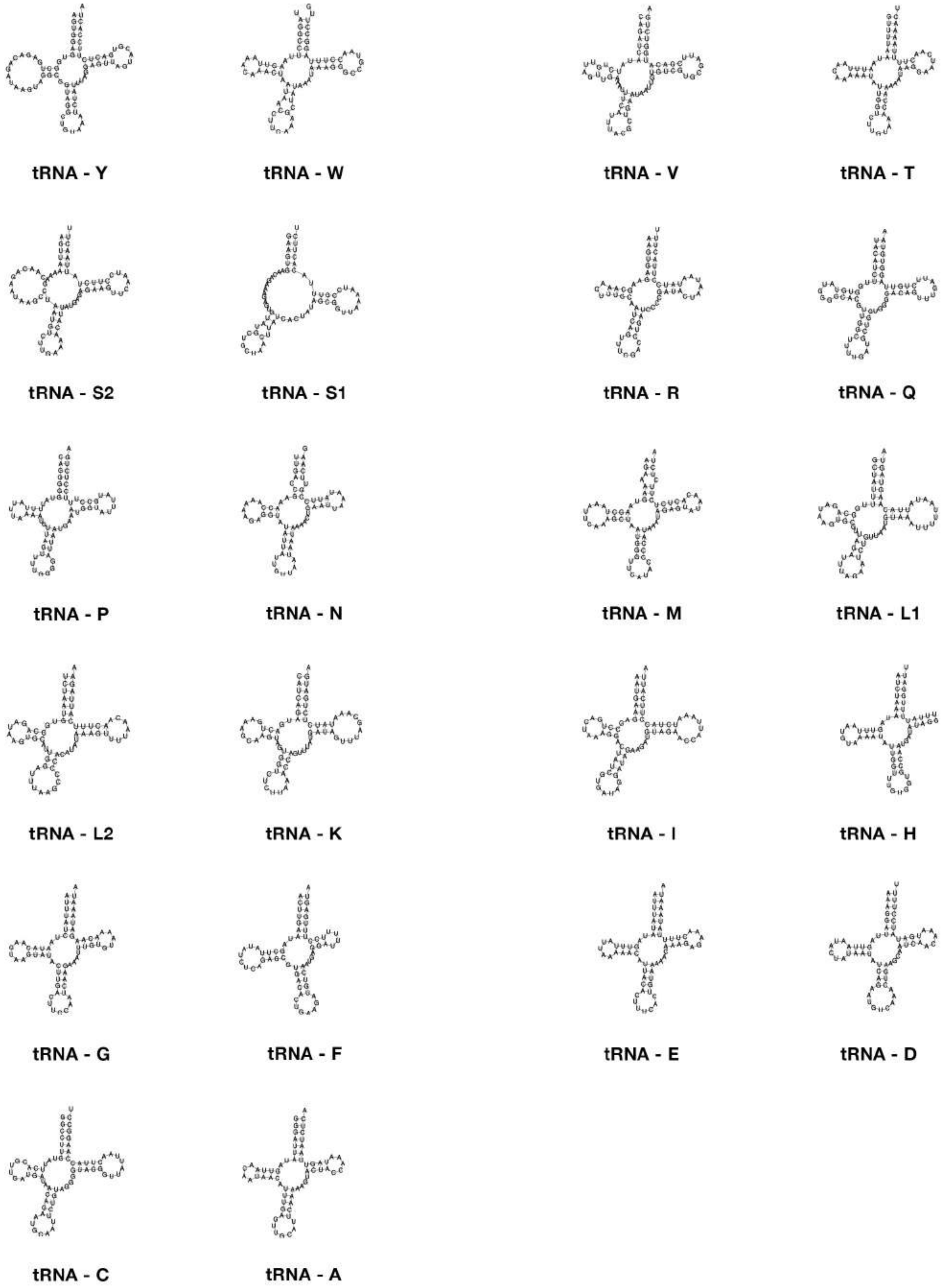

Reticulitermes lucifugus MK088051.1

Figure S2. Continued

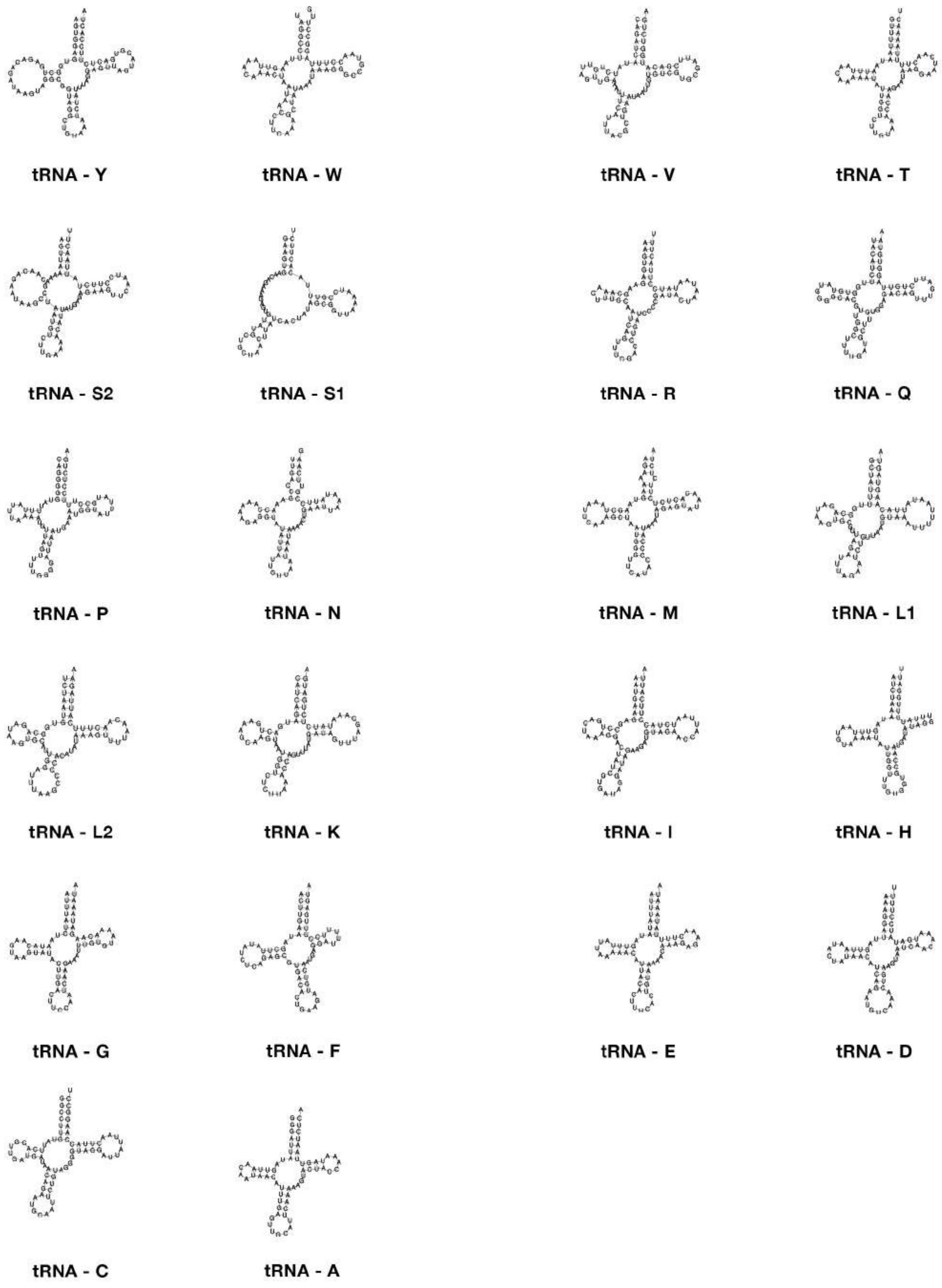

Reticulitermes lucifugus - intrafamiliar starting references

Figure S2. Continued

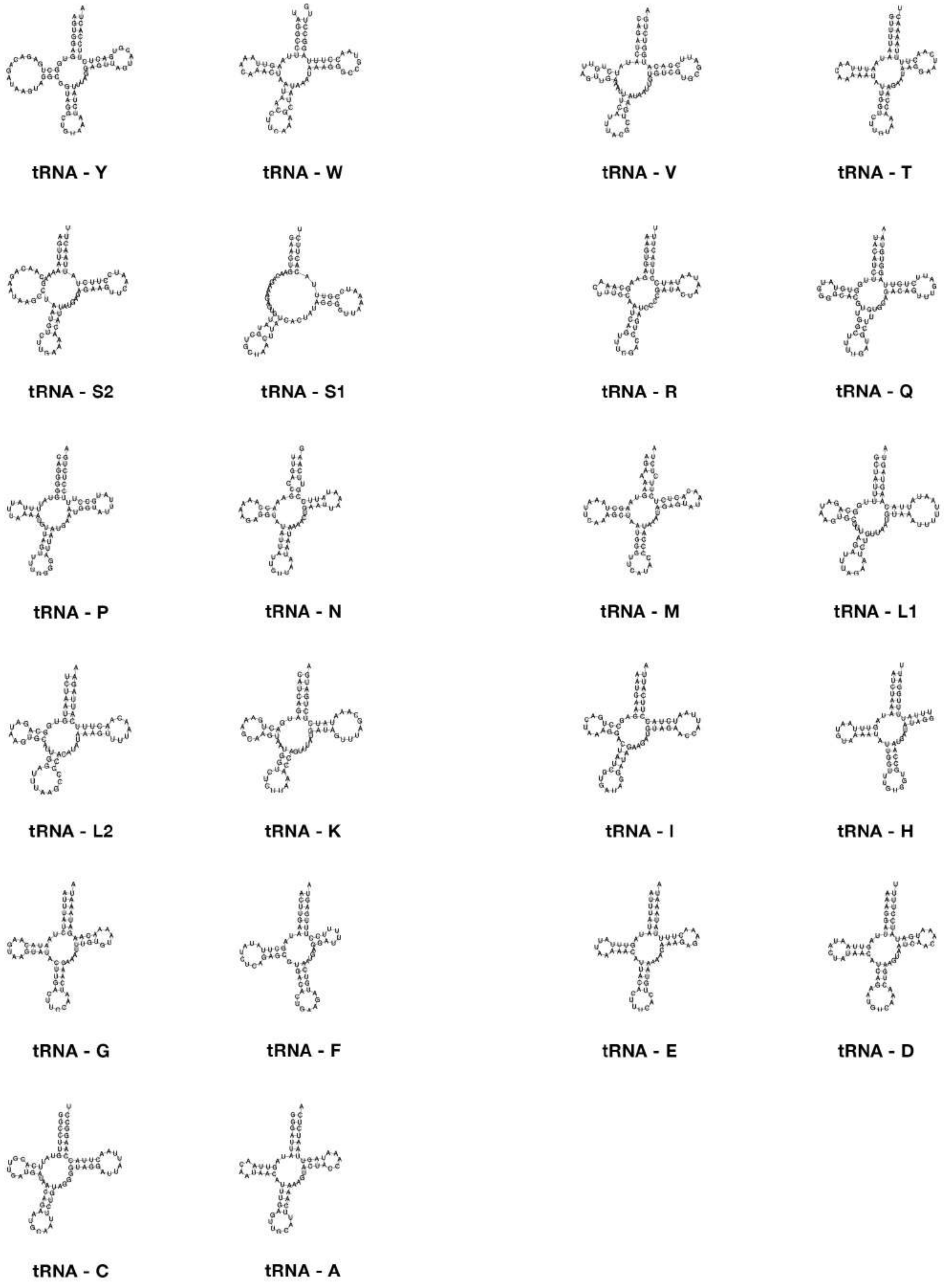

Reticulitermes lucifugus - congeneric starting references

Figure S2. Continued

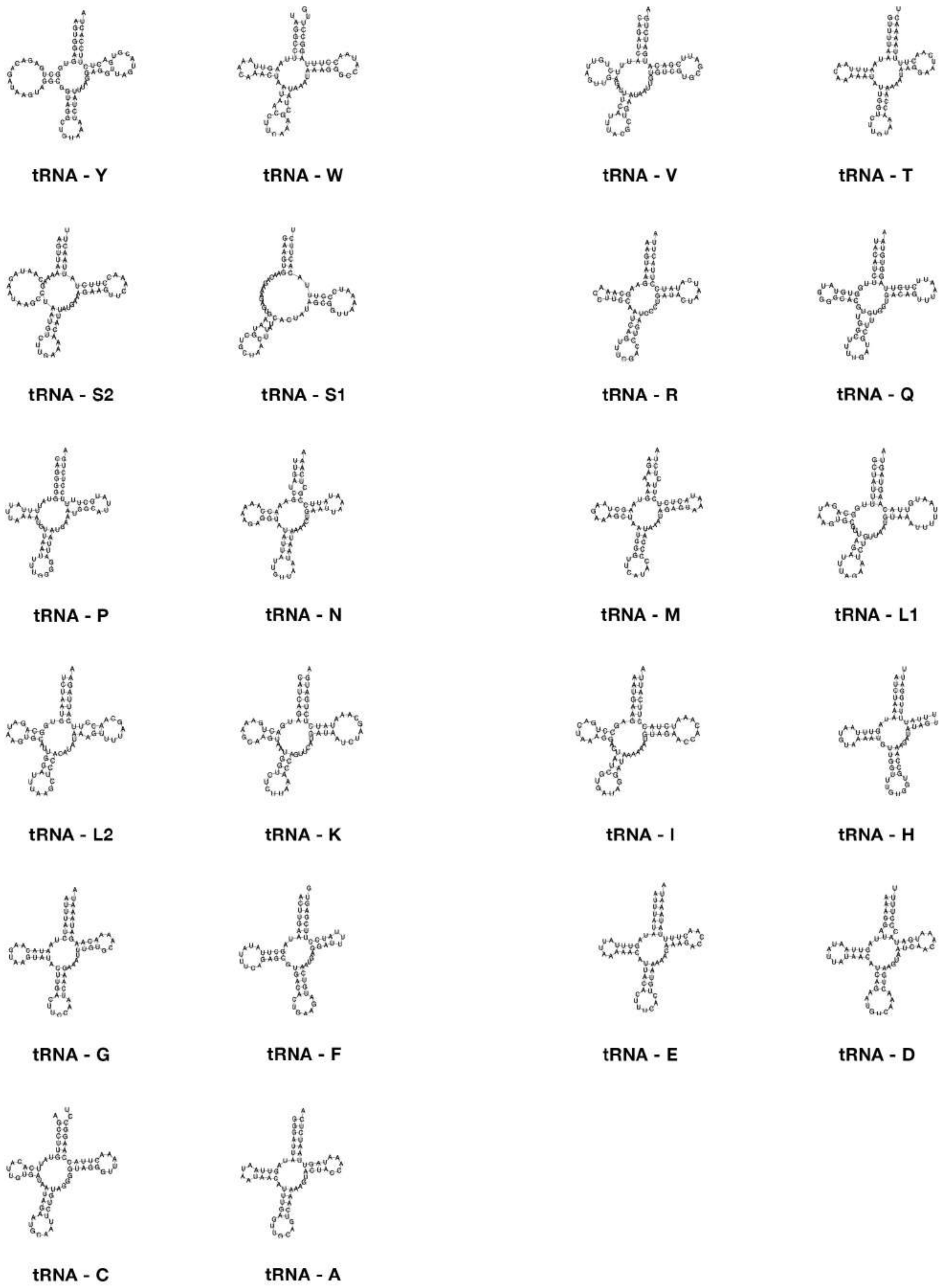

Reticulitermes speratus KY484910.1

Figure S2. Continued

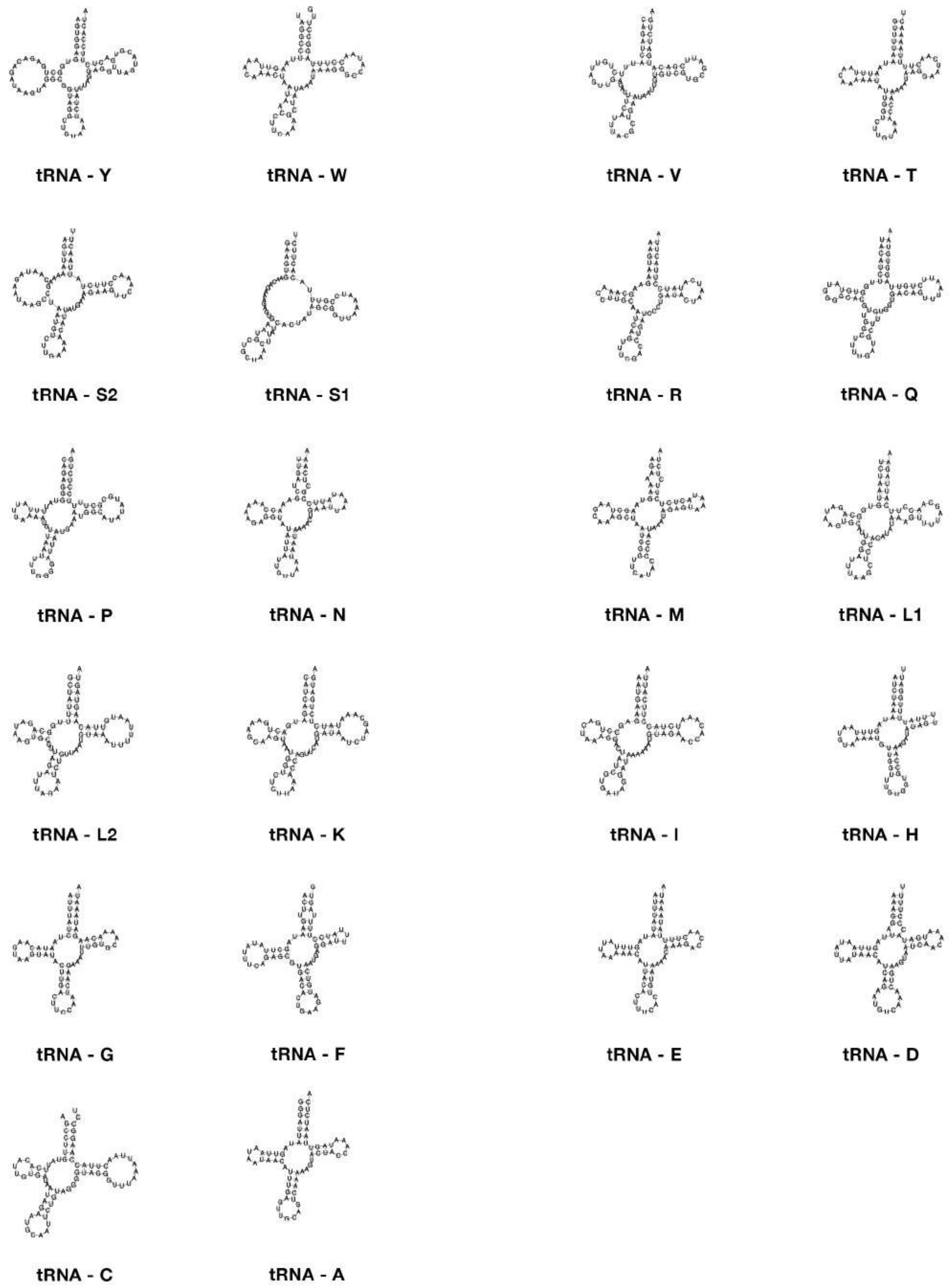

Reticulitermes speratus - intrafamiliar starting references

Figure S2. Continued

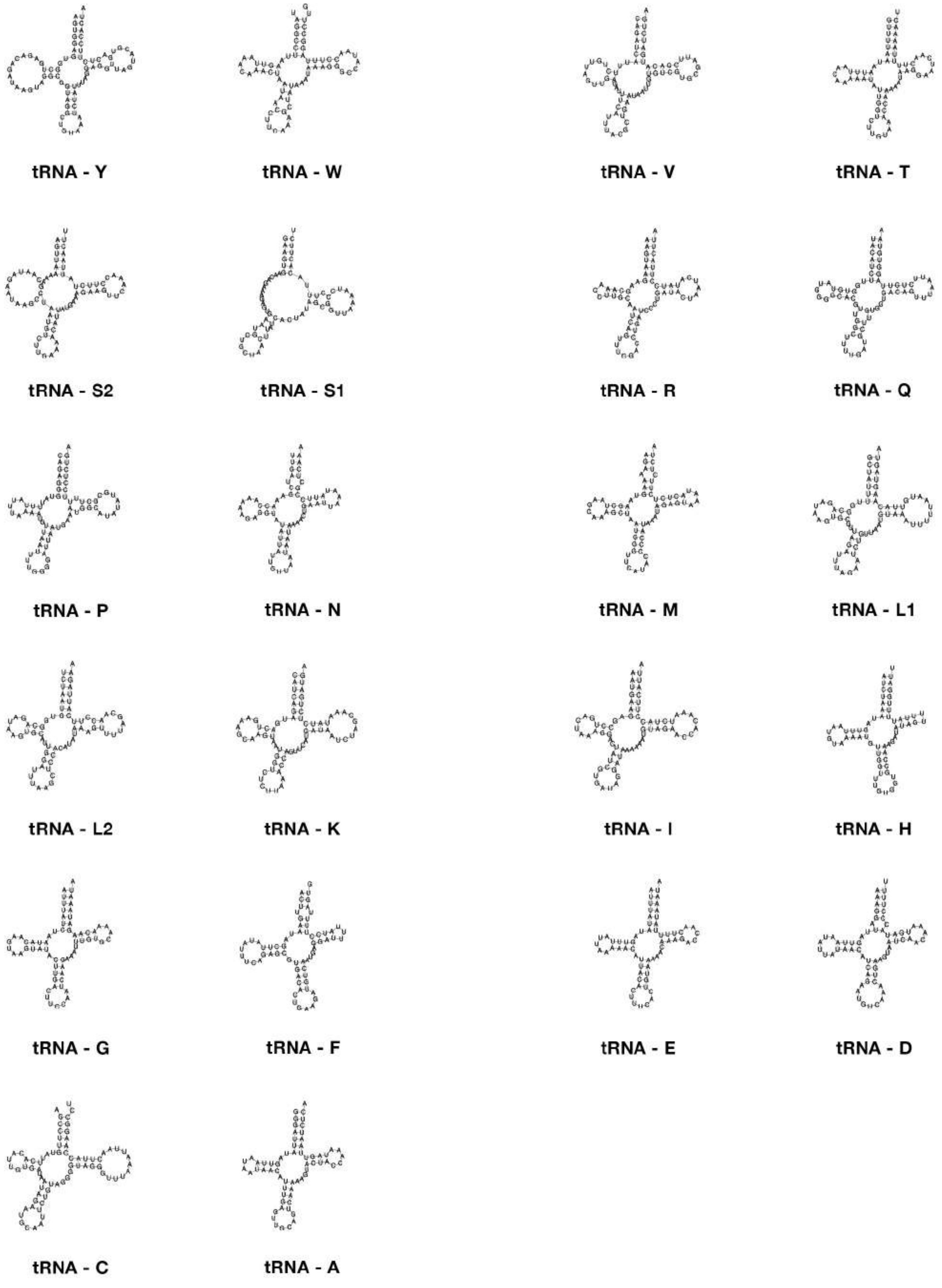

Reticulitermes speratus - congeneric starting references

Figure S3. Alignment of contigs reconstructed from RNA-Seq experiments using the reference with rearranged gene order, to the correct mitogenome sequence. Artificial rearrangements are indicated by shaded areas.

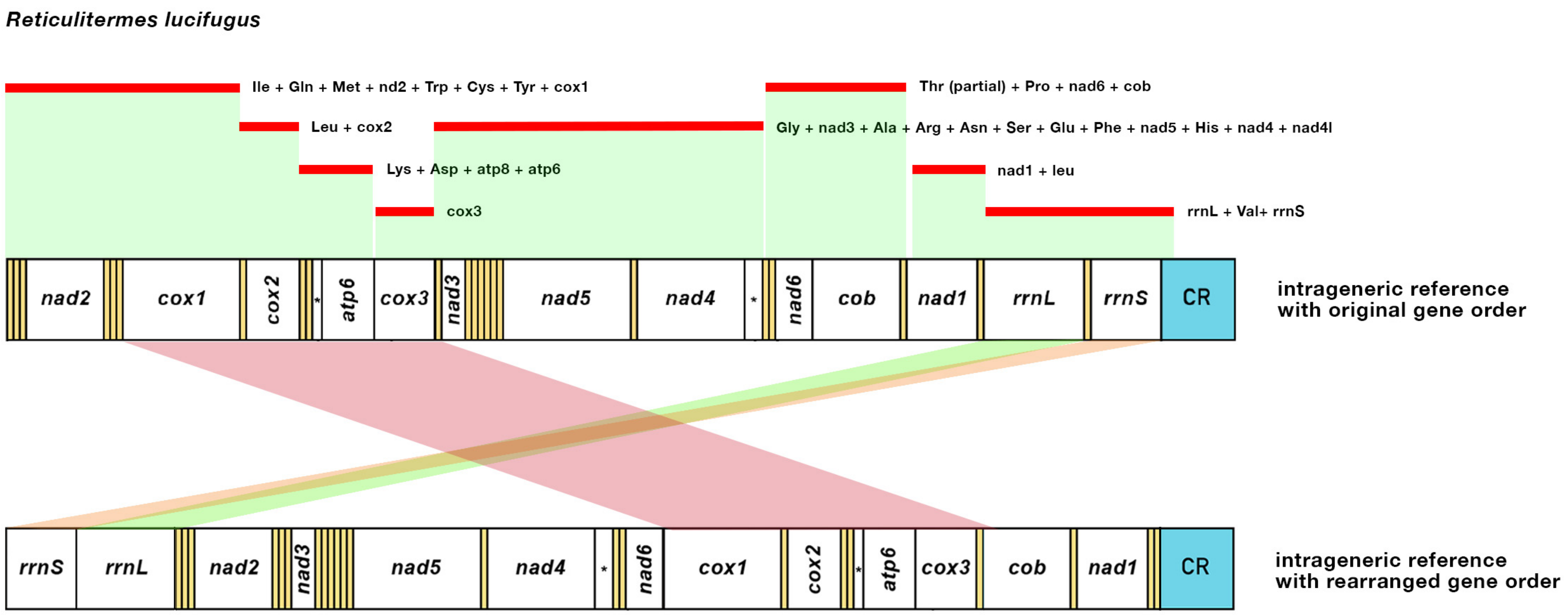

Figure S3. Continued

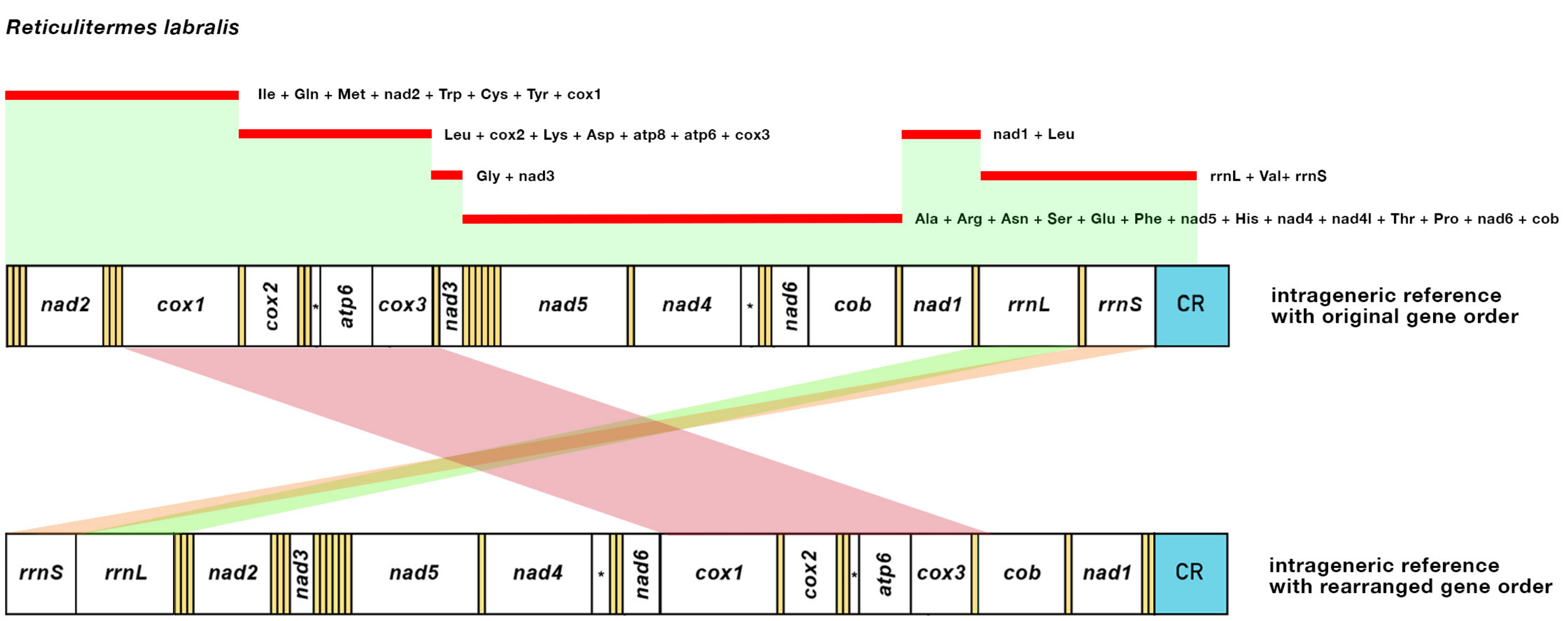

Figure S3. Continued

Reticulitermes speratus

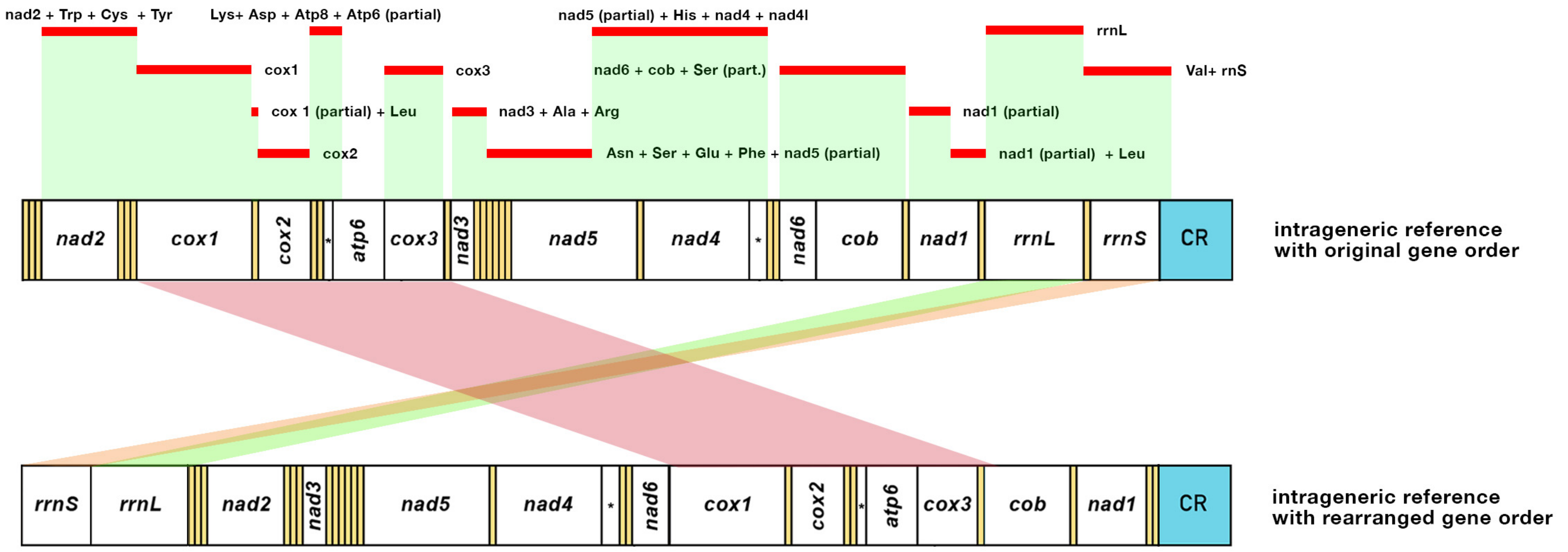

Figure S3. Continued

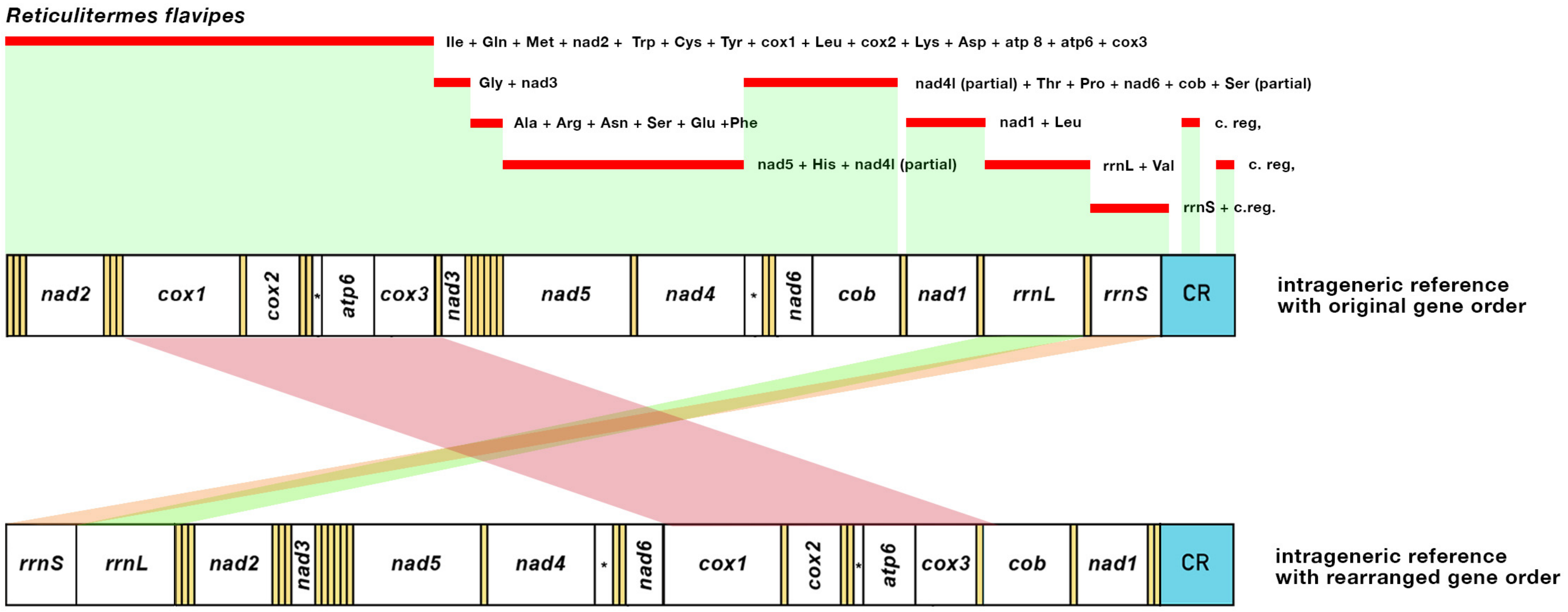

Figure S3. Continued

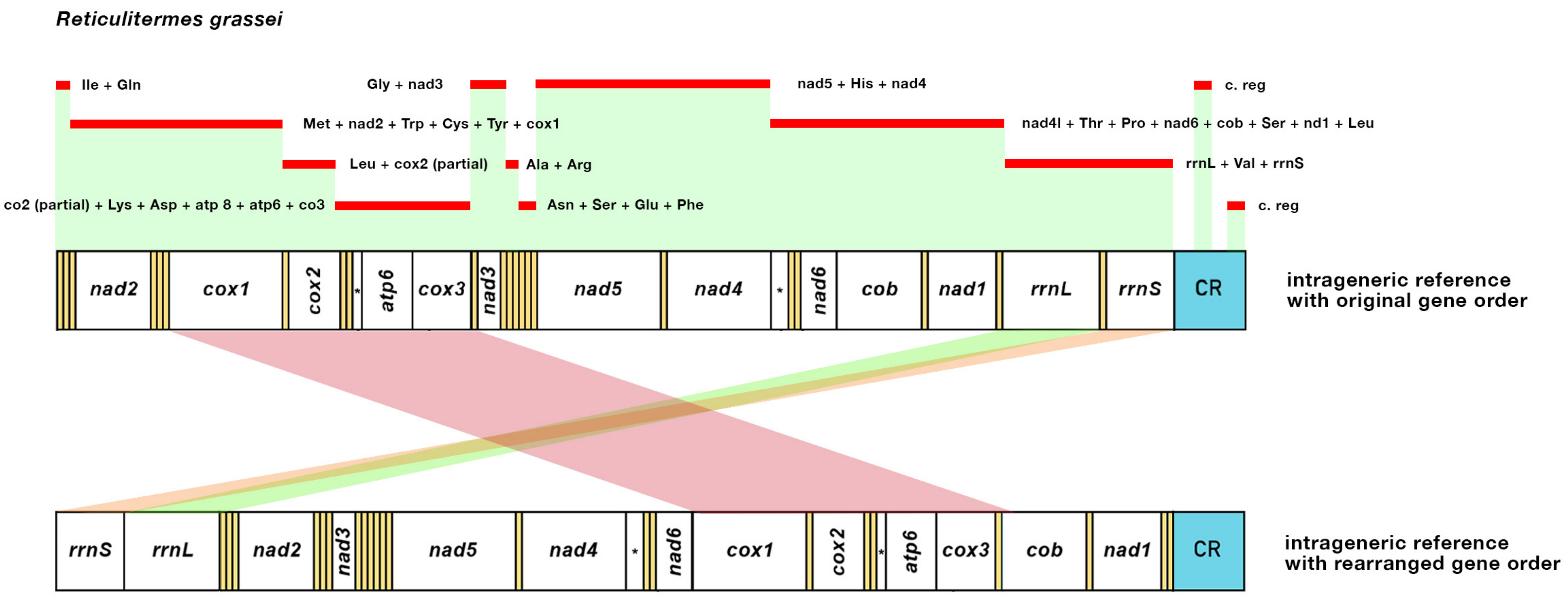

Figure S3. Continued

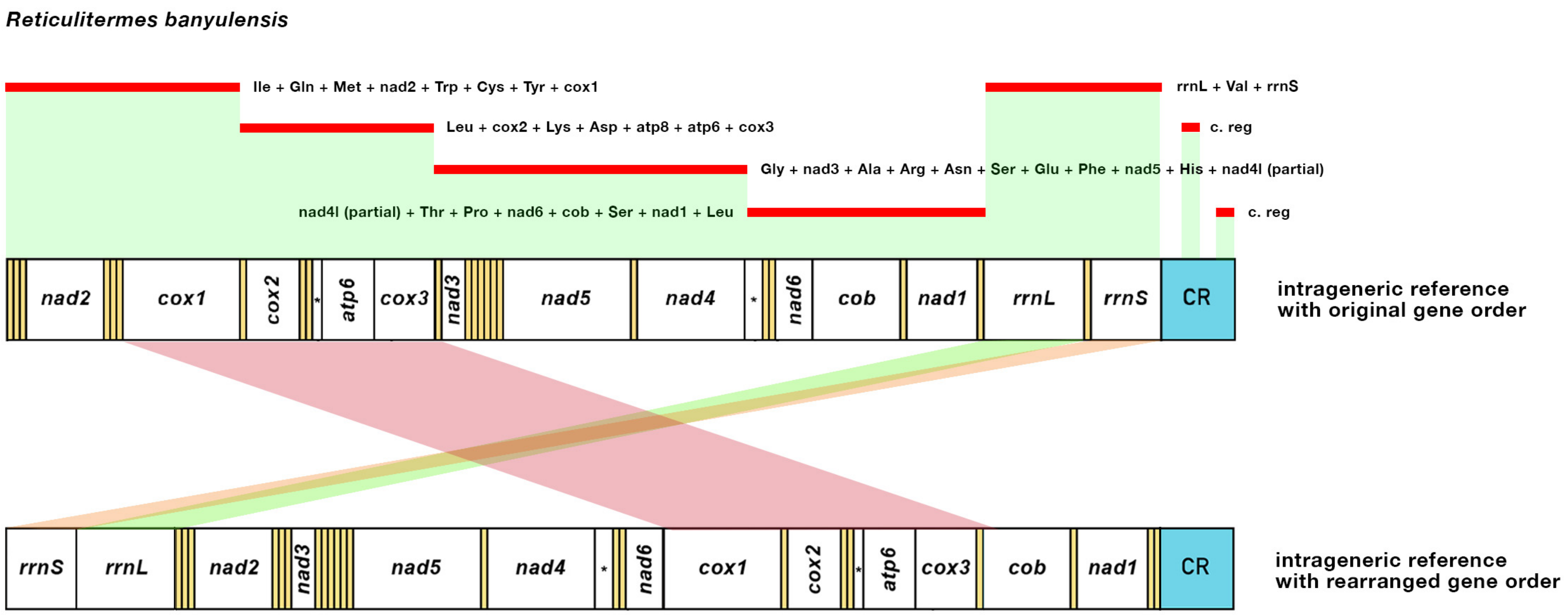

Supplement: Supplementary file 1 — Supplementary Information [file 41598_2019_51313_MOESM1_ESM.pdf]
